# Nanocellulose composite wound dressings for real-time pH wound monitoring

**DOI:** 10.1016/j.mtbio.2023.100574

**Published:** 2023-02-06

**Authors:** Olof Eskilson, Elisa Zattarin, Linn Berglund, Kristiina Oksman, Kristina Hanna, Jonathan Rakar, Petter Sivlér, Mårten Skog, Ivana Rinklake, Rozalin Shamasha, Zeljana Sotra, Annika Starkenberg, Magnus Odén, Emanuel Wiman, Hazem Khalaf, Torbjörn Bengtsson, Johan P.E. Junker, Robert Selegård, Emma M. Björk, Daniel Aili

**Affiliations:** a Laboratory of Molecular Materials, Division of Biophysics and Bioengineering, Department of Physics, Chemistry and Biology, Linköping University, SE-581 83, Linköping, Sweden; b Division of Materials Science, Department of Engineering Sciences and Mathematics, Luleå University of Technology, SE-971 87, Luleå, Sweden; c Center for Disaster Medicine and Traumatology, Department of Biomedical and Clinical Sciences, Linköping University, SE-581 85, Linköping, Sweden; d Division of Nanostructured Materials, Department of Physics, Chemistry and Biology (IFM), Linköping University, SE-58183, Linköping, Sweden; e Cardiovascular Research Centre, School of Medical Sciences, Örebro University, SE-70362, Örebro, Sweden

**Keywords:** Bacterial nanocellulose, Wound dressing, pH sensor, Infection, Mesoporous silica nanoparticles

## Abstract

The skin is the largest organ of the human body. Wounds disrupt the functions of the skin and can have catastrophic consequences for an individual resulting in significant morbidity and mortality. Wound infections are common and can substantially delay healing and can result in non-healing wounds and sepsis. Early diagnosis and treatment of infection reduce risk of complications and support wound healing. Methods for monitoring of wound pH can facilitate early detection of infection. Here we show a novel strategy for integrating pH sensing capabilities in state-of-the-art hydrogel-based wound dressings fabricated from bacterial nanocellulose (BC). A high surface area material was developed by self-assembly of mesoporous silica nanoparticles (MSNs) in BC. By encapsulating a pH-responsive dye in the MSNs, wound dressings for continuous pH sensing with spatiotemporal resolution were developed. The pH responsive BC-based nanocomposites demonstrated excellent wound dressing properties, with respect to conformability, mechanical properties, and water vapor transmission rate. In addition to facilitating rapid colorimetric assessment of wound pH, this strategy for generating functional BC-MSN nanocomposites can be further be adapted for encapsulation and release of bioactive compounds for treatment of hard-to-heal wounds, enabling development of novel wound care materials.

## Graphical abstract

**Figure:**
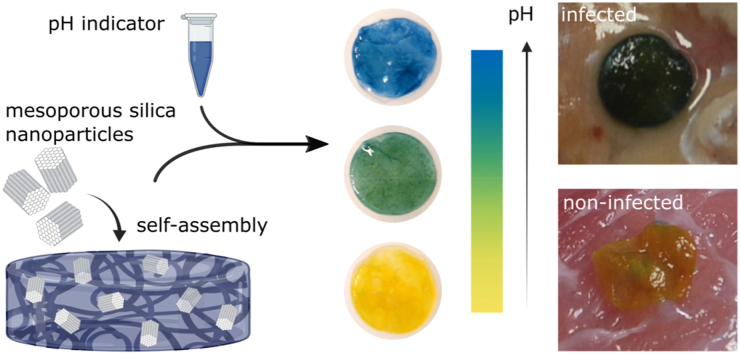

## 1 Introduction

Wound infections can result in serious complications for all types of wounds, including surgical wounds and burns [1,2], with increased risk of sepsis [3,4] and delayed healing as a consequence [1]. If the wound has not healed within 6 weeks to 3 months or is recurring, it is referred to as a non-healing, or chronic, wound [5,6]. Chronic wounds pose a great burden on the healthcare systems all over the world and result in reduced quality of life for millions of patients [7,8]. These non-healing wounds give rise to persistent pain, distress, anxiety, and chronic morbidity and result in prolonged hospital stays and increased mortality [9]. In the United States, 3–4% of the population >65 years of age have open wounds [10]. Cost projection for wound treatment in the US is in the range from $28.1 to $96.8 billion and are expected to rise sharply globally as a result of an aging population, increasing obesity rates and incidence of diabetes [11]. Moreover, with increased antibiotic resistance among pathogens, better diagnostic methods and new treatment options for chronic wound infections are urgently needed [12].

Wound healing is a complex process and involves a multitude of different factors important for optimal healing, including clearing of foreign bodies and pathogens by the immune system and tissue remodeling mediated by resident cells. In medically compromised patients, an elevated inflammatory response results in reduced tissue reconstitution, which further contributes to the chronification of wounds [13]. For a chronic wound to heal, the infection must be controlled and ideally eradicated. This is commonly achieved using a combination of debridement of dead tissue, topical antiseptics, and systemic administration of antibiotic agents. Surgical or mechanical debridement can, however, damage healthy tissue and retard the healing process while systemic antibiotics increase the risk of antibiotic resistance in pathogens [14]. Additionally, bacterial infections often lead to extensive biofilm formation, which protects the bacteria and makes them less susceptible to antibiotics [15].

Wound infections are generally diagnosed by visual inspection, which is both subjective and requires an infection to have progressed to a level where clinical symptoms such as redness, swelling, pus and pain are clearly present [1,16]. At this stage, the microbial load is high, and the infection can be very difficult to treat. Wound swabs followed by culture are sometimes used to identify the causative organism, which is time-consuming and subject to variability depending on differences in clinical practice [16,17]. Early-stage detection and diagnosis of wound infections can enable a more efficient treatment and reduce the risks of developing non-healing wounds [5]. Several different sensor designs have been investigated for early detection of wound infections and sepsis, including nanoplasmonic-based point-of-care devices for rapid detection of procalcitonin and C-reactive protein [18], wearable electrical [19], and optical [20] temperature sensors, as well as colorimetric [21], potentiometric [22], and fluorometric [23] pH sensors.

Whereas the pH of intact and non-infected skin is slightly acidic and typically varies between 4 and 6 [24], the pH values of chronic wounds are typically in the range of 7–9 [[24], [25], [26]]. Slower healing occurs in both acute and chronic wounds with an elevated alkaline pH, compared to wounds with a pH closer to neutral [27,28]. Moreover, infections tend to trigger an increase in wound pH [25,29,30], which is often manifested before other clinical symptoms [31]. Sensors monitoring wound pH could hence potentially enable assessment of wound status and detection of early-stage infections [24,32]. However, clinical translation and implementation of wound pH sensors requires that the sensors are robust, reliable and can produce a distinct sensor readout without the need for elaborate equipment. In addition, removal of the wound dressing to monitor biomarkers, such as pH, increase risks of interfering with the healing process and introducing new pathogens. Integration of the sensors in the wound dressing materials is consequently central and could facilitate handling and enable close contact between the sensors and the wound.

Development of new advanced wound dressing materials [33,34] and improved strategies for functionalization of the dressings [35,36] has paved the way for wound diagnostic strategies that limits the impact on wound healing [37]. Akbari and co-workers have reported a colorimetric pH-responsive alginate-based wound dressing obtained by loading pH indicators in ion-exchange beads that were trapped in the hydrogel matrix by electrostatic interactions [38]. Alginate is a widely used material for wound dressings [39,40], however, the pH-responsive dressings were not mechanically robust and sensitive to dehydration. Whereas the former complicates handling, the latter can result in very high-water vapor transmission rates (WVTR) which has a negative impact on wound healing [40]. Liu et al. addressed these problems by reinforcing alginate hydrogels with a covalently cross-linked second network of polyacrylamide, resulting in higher tensile strength and improved WVTR [41]. The hydrogels were further modified by covalent conjugation of the pH-responsive dye phenol red to the polymers. However, in addition to the complex synthesis, the colorimetric response showed poor contrast to wound tissues, making the readout difficult. To improve contrast, Gamerith et al. developed a method for immobilizing the pH-responsive dye bromocresol purple (BCP) to cellulose-based materials, such as filter paper and cotton [42]. BCP changes color from yellow to blue with increasing pH, a color transition that could be clearly visible in wounds. However, the conjugation of the dye to cellulose required heating of the materials to 120 ​°C, which is not compatible with state-of-the-art hydrogel wound dressings. An alternative strategy was presented by Khademhosseini and co-workers, where a pH-responsive material based on mesoporous polyester beads was loaded with the pH sensitive dye brilliant yellow and embedded in alginate hydrogel microfibers [43]. The fibers could subsequently be adsorbed on conventional wound dressings, such as fiber bandages. Although the colorimetric contrast of the dye used was very poor in a wound environment, the concept of loading the dye in a carrier particle is benign and allows for further optimization of the sensor response.

**Fig. 1:**
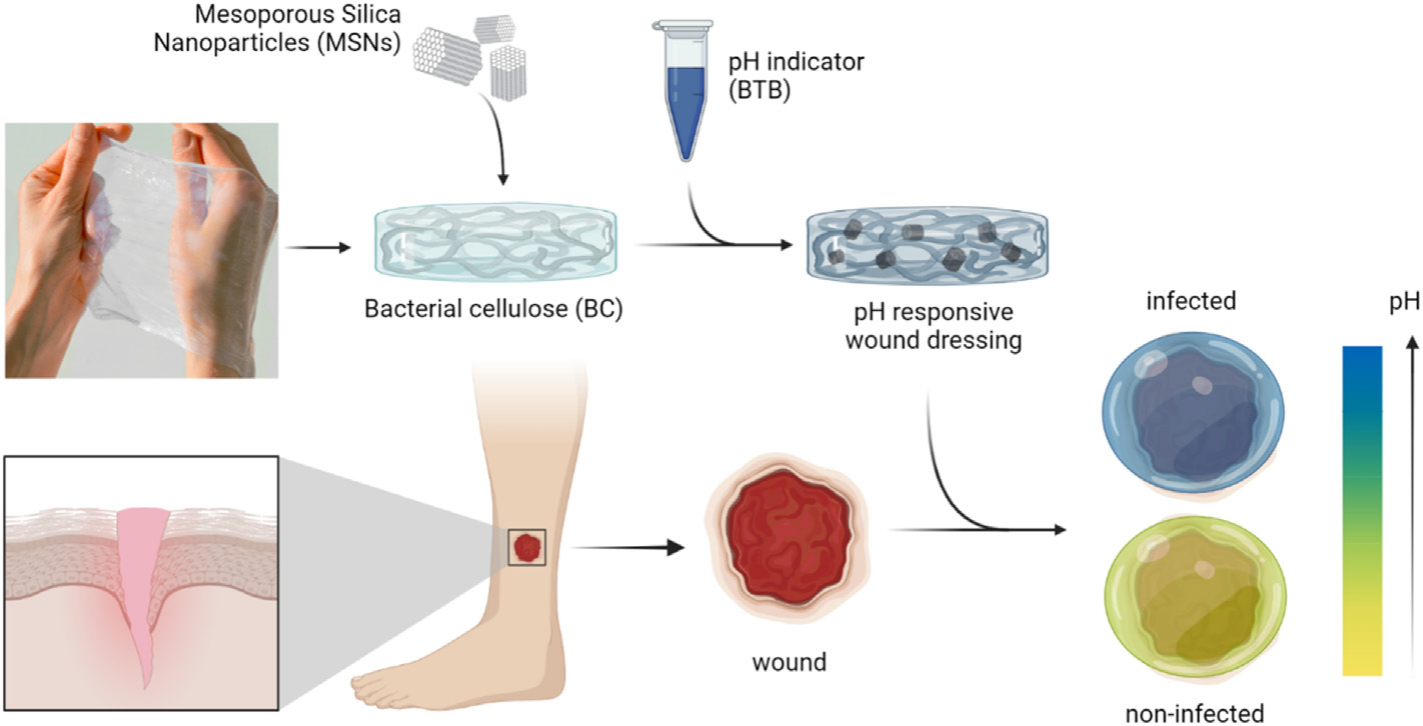
pH-responsive nanocomposite wound dressings were obtained by impregnating bacterial nanocellulose with mesoporous silica nanoparticles loaded with a pH-responsive dye.

Here, we show a pH-responsive composite nanocellulose-based wound dressing material (pH@BC) that allows for rapid high contrast visual colorimetric readout of wound pH. The wound dressings were fabricated from bacterial nanocellulose (BC), impregnated with mesoporous silica nanoparticles (MSNs) that were loaded with a pH-responsive dye (Fig. 1). BC is a highly hydrated material comprised of a complex three-dimensional (3D) arrangement of nanocellulose fibrils, which form a tight mesh that mimics the structure of the extra cellular matrix (ECM). The nanoscale porosity prevents penetration of bacteria but allow for diffusion of liquids, gases, and macromolecules. The large water holding capacity, and good mechanical properties of BC facilitate handling and promotes a moist wound microenvironment that favors healing. The high conformability allows for close contact between the wound tissue and the dressing which relieves pain and stops bleeding [44,45], and can also potentially allow for sensitive detection of biomarkers when integrating sensor elements in the BC.

We have recently demonstrated that BC-based wound dressings can be functionalized with a wide range of metal nanoparticles (NPs) by self-assembly, resulting in nanocomposites with unique optical and antimicrobial properties [46]. This process is driven by short range van der Waals interactions between the NPs and the BC. By tuning the interactions and conditions for the self-assembly process, large quantities of well-defined NPs could be adsorbed. Here, we have modified and optimized this approach for adsorption of MSNs in BC wound dressings as a carrier for pH-responsive dyes. MSNs have a large specific surface area [47] and have been utilized in numerous applications, such as purification [48,49], catalysis [47] and drug delivery [[50], [51], [52]]. Biomedical applications are facilitated by their low toxicity, high stability [53,54], well-defined pore structure [55] and tunable surface chemistry [56,57]. Even with large quantities of incorporated MSNs, the BC dressings showed excellent mechanical properties, water retention, and vapor transmission rate. The MSNs were further loaded with the pH indicator bromothymol blue (BTB). BTB shows a color shift from yellow to green to blue in the pH range 5.5–8. The colors are discrete at low pH but change to a high contrast color when pH increases above 6, corresponding to a relevant range for detection of wound infections. The MSNs employed here, SBA-15, are optically transparent and about 400 ​nm in size and are characterized by ordered hexagonal nanopores [55]. The high surface area of the nanocomposites allowed for loading of large quantities of BTB in the dressings, which facilitated readout of wound pH. Without the MSNs, negligible amount of BTB were retained in the BC dressings. This pH-responsive nanocomposite hence combines excellent wound dressing material properties with rapid and non-invasive visual pH monitoring for wound assessment and early detection of infections.

## 2 Materials and methods

*General:* BC produced from *Komagataeibacter xylinus* was obtained from S2Medical AB (Linköping, Sweden). All chemicals were obtained from Merck KGaA (Darmstadt, Germany) and used without further purification unless otherwise noted.

*MSN Synthesis:* MSNs were synthesized using a protocol from Björk et al. [55] Shortly, 2.4 ​g of Pluronic P123 (PEO_20_PPO_70_PEO_20_, M_n_ ∼5800) and 28 ​mg ammonium fluoride (>98%) (Honeywell Fluka, Charlotte, NC, USA) were dissolved in 80 ​mL 1.84 ​M HCl (from ≥37%) using magnetic stirring at 20 ​°C. A solution of 5.5 ​mL of tetraethyl orthosilicate (98%) and 1.0 ​mL of heptane (99%) were premixed and added to the micelle solution. The mixture was stirred for 4 ​min and then kept under static conditions over-night at 20 ​°C. The solution was transferred to a PTFE flask for hydrothermal treatment at 100 ​°C for 24 ​h. The MSN were collected by filtration and washed with deionized water. To remove surfactants, MSN were calcined at 550 ​°C for 5 ​h with a ramp of 2 ​°C/min.

*Nitrogen Physisorption Measurements:* Specific surface area was calculated from nitrogen physisorption isotherms obtained using an ASAP 2020 (Micromeritics Instrument Inc., Norcross, GA, USA) operated at −196 ​°C. Prior to the measurements, the samples were degassed at 110 ​°C for 6 ​h. Specific surface was calculated using the BET-method on the adsorption isotherm at P/P_0_ ​= ​0.07–0.18.

*MSN Immobilization in BC:* BC dressings were cut into 6 ​mm discs with a thickness of roughly 0.2 ​× ​10^−3^ ​m using a biopsy punch. The BC discs were rinsed in MilliQ water before any further treatment. The pH-responsive wound dressings were synthesized by incubating the BC discs in 0.5 ​mL suspension of 5 ​mg/mL MSN in MilliQ water for 5 days on an orbital shaker. After the incubation, the BC discs were rinsed in MilliQ water and placed in 200 ​μL 2 ​mM BTB solution and incubated overnight. The discs were then transferred directly from the BTB incubation to a solution of 200 ​μL PEI 4 ​mg/mL, 1 ​M NaCl, pH 5.5 in MilliQ water and incubated for 1 ​h. The discs where then repeatedly rinsed in MilliQ water.

*Adsorption of BTB in Suspended Mesoporous Silica Nanoparticles:* MSNs were suspended in MilliQ water by vortexing for several minutes and ultrasonication for 1 ​min. Particles and supernatant were then separated using centrifuging with a micro centrifuge. For every 10 ​mg of MSN, 200 ​μL of BTB (2 ​mM pH 8) was added, particles were resuspended by vortexing and incubated overnight. Particles were then centrifuged, and the supernatant discarded. For every 10 ​mg of MSN, 200 ​μL PEI (4 ​mg/mL, 1 ​M NaCl, pH 5.5) (ICN Biomedicals Inc., Aurora, OH, USA) was added. Particles were resuspended and incubated in the PEI solution for 1 ​h. Particles were washed with MilliQ water by centrifuging and replacing the supernatant with MilliQ water.

*Effect of PEI Capping on BTB Release:* MSNs were incubated in BTB solution (2 ​mM, pH 8) according to the protocol described previously. Following BTB loading, MSNs were centrifuged, and supernatant removed. One part of loaded MSN was further functionalized with PEI according to the protocol described above. MTA buffer was prepared with 50 ​mM (4- morpholineethanesulfonic acid), 100 ​mM TRIS (2-amino-2- (hydroxymethyl)-1,3-propanediol), and 50 ​mM acetate. Particles were split in 5 ​mg aliquots and incubated in 1.5 ​mL of MTA buffer at pH 5, 7 or 9. BTB release was quantified using a microplate reader (Tecan Infinite M1000 Pro, Tecan Austria GmbH, Grödig/Salzburg, Austria). Measurements were taken in triplicates.

*Effect of MSN on BTB Loading and Retention:* BC and BC-MSN dressings were incubated overnight in BTB solution (2 ​mM, pH 8). Excess BTB was carefully removed by patting with dry tissue paper, prior to submerging the membranes in 200 ​μL PEI (4 ​mg/mL, 1 ​M NaCl, pH 5.5). Extinction spectra were recorded with a microplate reader (Tecan Infinite M1000 Pro, Tecan Austria GmbH, Grödig/Salzburg, Austria). The samples were subsequently washed three times by incubation in 250 ​μL of MTA buffer (pH 9) for 30 ​min. Extinction spectra of the membranes were then acquired to quantify BTB retention.

*Effect of BTB Incubation Concentration on Dressing Color Intensity:* Four BC-MSN dressings were transferred to 200 ​μL, pH 8, BTB-solution of 2 ​mM, 1 ​mM, 0.5 ​mM and 0.1 ​mM in MilliQ water and left to incubate overnight. The MSNs were then capped with PEI (4 ​mg/mL, 1 ​M NaCl pH 5.5, ICN Biomedicals Inc., Aurora, OH, USA) and rinsed. Spectra were acquired using a microplate reader (Tecan Infinite M1000 Pro, Tecan Austria GmbH).

*Wound Dressing Response to* pH: The pH-responsive dressings were subjected to 100 ​μL 100 ​mM citrate buffer at pH 5.5, 6, 6.5 or PBS pH 7, 7.5, 8, 8.5, and incubated for 45 ​min ​+15 ​min on an orbital shaker. UV–vis spectra of the dressings were recorded using a microplate reader (Tecan Infinite M1000 Pro, Tecan Austria GmbH).

*Spatial* pH *Detection:* A pH-responsive dressing was placed on a glass slide and excess water was removed. A droplet of 1 ​μL of 0.1 ​M HCl was added to the left side of the dressing and a droplet of 1 ​μL of 0.1 ​M NaOH was added to the right side.

*Scanning Electron Microscopy:* Dressings were dried overnight in a desiccator cabinet or by freeze drying. Dressings were then placed on conducting carbon tape and sputter coated with platinum for 10 ​s. Images were acquired using a Zeiss LEO Gemini 1550 (Carl Zeiss, Oberkochen, Germany) with an InLens detector. Acceleration voltage was set to 3 ​kV.

*Transmission Electron Microscopy:* MSNs were suspended in ethanol and added to a hollow carbon grid. Samples were imaged using a FEI Tecnai G2 TF 20 UT (FEI Company, Hillsboro, OR, USA) microscope operated at 200 ​kV.

*X-ray Microtomography (XRT):* The structure of BC and BC-MSN (incubated in 5 ​mg/mL of MSN suspension for 1 day and 5 days) were 3D reconstructed after freeze-drying using a Zeiss Xradia 510 Versa (Carl Zeiss, Dublin, CA, USA) with a 20 ​× ​objective, with a field of view of 0.56 ​mm and voxel size of 0.56 ​μm. Samples sized approximately 1.5 ​mm^3^ were scanned with an X-ray tube voltage of 50 ​kV, output power of 4 ​W, and no X-ray filters. A total of 2401 projections with an exposure time of 6 ​s resulted in a total scan time of 6 ​h. Reconstruction was performed using filtered back-projection with the Zeiss Scout-and-Scan Reconstructor software (version 11.1) and Dragonfly Pro Software (ORS) (Carl Zeiss, Dublin, CA, USA) was used for the 3D visualization of the structure.

*Thermogravimetric Analysis:* Thermogravimetric analysis (TGA) was performed to measure MSN wt % in BC. BC-MSN dressings were prepared as previously described, by incubating BC dressings in a suspension of 2, 5 and 10 ​mg/mL MSN for 1, 3, 5 and 7 days under constant shaking (100 min^-1^). Dressings were then rinsed in MilliQ water and dried before analysis. TGA curves were obtained using a STA 449 ​C Jupiter Thermo-microbalance (Netzsch-Gerätebau GmbH, Selb, Germany). For this study, 10 to 15 samples were used. Samples were heated in an open alumina crucible from 25 to 550 ​°C under air atmosphere at heating rate of 10 ​°C/min. The mass of each sample was in the range of 4–10 ​mg, and weight loss was recorded from 120 to 550 ​°C. Data acquisition was performed with Proteus Analysis software (Netzsch-Gerätebau GmbH, Selb, Germany).

*Stability test of BC-MSN:* The spontaneous detachment of MSNs from BC-MSN dressings was evaluated by incubating BC-MSNs (ø 6 ​mm, n ​= ​10) in 200 ​μL 10 ​mM PBS for 3 days at room temperature under shaking. Following incubation, UV–vis spectra of the incubation solutions were acquired using a microplate reader (Tecan Infinite M1000 Pro, Tecan Austria GmbH, Grödig/Salzburg, Austria) to determine the concentration of detached MSN.

*Water Content:* Water content of BC-MSN dressings was measured to investigate the impact of MSN on the water uptake capacity of BC. The dry weight of the dressings was determined post dehydration by TGA with a STA 449 ​C Jupiter Thermo-microbalance (Netzsch-Gerätebau GmbH, Selb, Germany). Samples were heated in an open alumina crucible from 25 to 110 ​°C under air atmosphere at heating rate of 1 ​°C/min. The mass of each sample was in the range of 30–75 ​mg. Percent water content was calculated according to the following equation:

(1) $$\%\;water\;content=\frac{\left(W_{w}-W_{d}\right)}{W_{d}}\times 100$$

where $W_{w}$ and $W_{d}$ are the weights of the wet and dry sample, respectively. The experiments were performed in triplicates.

*Compression Rheology:* Measurements were carried out on a Discovery HR-2 rheometer (TA instruments, New Castle, DE, USA) using an 8 ​mm parallel plate geometry. Measurements were carried out at a temperature of 25 ​°C, regulated by a Peltier element, using BC discs with a diameter of 8 ​mm. Before each measurement, the upper plate was lowered in proximity to the sample, and extra MilliQ water was added in the gap. Water flow in the radial direction was unconstrained during compression. Prior to the measurements, the linear viscoelastic region (LVR) of the materials was identified and appropriate frequency and oscillation strains (1 ​Hz and 0.01%, respectively) were selected within this region for oscillatory rheology measurements (data not shown). The samples were iteratively compressed and allowed to relax at different gap heights. The axial compression was performed at a speed of 5 ​μm/s until axial force levels of 0.1, 0.5, 1, 2, 4 and 6 ​N were achieved to assess the material properties in the out-of-plane direction. After every compression step, gap height was maintained constant and the sample was allowed to relax for 2 ​min, while subjected to small amplitude oscillatory deformations (SAOS), to measure the viscoelastic properties. The axial force was recorded throughout the whole measurement and plotted as a function of time to measure the characteristic relaxation at 2 ​min. Storage (G′) and Loss moduli (G″) were recorded during the oscillatory rheology steps, and plotted as a function of time, gap, and dry content. All samples were run in quadruplicates and representative curves are presented.

*Tensile Testing:* Mechanical properties of BC, BC-MSN and pH@BC were measured using a Shimadzu Autograph AG-X universal tensile testing machine (Shimadzu Corp., Kyoto, Japan) equipped with a load cell of 1 ​kN. Tests were performed at a strain rate of 2 ​mm/min with a gauge length of 20 ​mm. Samples were formed into strips of 6 ​mm in width and 80 ​mm in length and kept in MilliQ water prior to testing. Average values are based on at least five measurements per sample.

*Water Vapor Transmission Rate (WVTR):* WVTR of the dressings was calculated according to SS-EN 13726-2 standard with minor modifications. Briefly, 12 ​mm circular samples were positioned on the mouth of cylindrical glass vials containing 3 ​mL of deionized water with a cap with a hole with 64 ​mm^2^ orifice area. The system was sealed with paraffin wax to prevent moisture loss. Vials were incubated at 37 ​°C in 32% RH for the duration of the test. Evaporation of water through the dressings was determined by periodic weightings over a period of 3 days. Weight loss data were fitted by linear regression excluding data points corresponding to the first 4 ​h, during which the weight loss was mainly driven by the drying of the exterior part of the dressing. Weight loss data were normalized against dressingh area and time, allowing calculation of the WVTR (g/m^2^/day). Five samples were tested per each condition.

*Water Retention Rate (WRR):* Dressings were incubated in MilliQ water for 24 ​h prior to testing. Hydrated dressings were blotted with wet laboratory paper to remove surface water and allowed to dry at room temperature. Mass loss was monitored at 30 ​min intervals, until drying. Successively, the dressings were completely dried in an oven at 80 ​°C to determine the dry weight. WRR was calculated according to the following equation:

$$WRR=\left[\frac{W_{t}-W_{d}}{W_{w}-W_{d}}\right]*100\;\%$$

where $W_{t}$ is the instantaneous weight, $W_{w}$ is the initial weight of the hydrated sample and $W_{d}$ is the weight of the dry sample. Samples were measured at least in quintuplicates.

*Cytocompatibility Evaluation on Primary Human Keratinocytes and Fibroblasts:* Cytocompatibility was evaluated using healthy human cells isolated from skin samples of healthy patients undergoing plastic surgery at Linköping University Hospital, Sweden. Experiments were carried out under ethical approval from the Swedish Ethical Review Authority (2018/97/31). Keratinocytes were isolated from the epidermis by removing subcutaneous fat and incubating the remaining skin in Dulbecco's Modified Eagle's Medium (DMEM; Gibco Thermo Fisher Scientific, Paisley, UK) containing 25 U/mL dispase (Gibco Thermo Fisher Scientific) for 18 ​h at 4 ​°C. The epidermis was dissected and enzymatically digested by incubation in 4 ​mL of 0.02% versene and 0.1% trypsin for 15 ​min at 37 ​°C, 5% CO_2_, and 95% humidity, while being vortexed every 2 ​min. The solution was centrifuged at 365 ​g for 5 ​min, the pellet was resuspended in 15 ​mL of keratinocyte medium and seeded in a 75 ​cm^2^ cell culture flask (Falcon, Corning Inc, Corning, USA) until confluency. To isolate fibroblasts, dermis was cut into 1 ​× ​3 ​mm^2^ pieces and placed in DMEM with 165 U/mL collagenase (Gibco Thermo Fisher Scientific, Paisley, UK) and 2.5 ​mg/mL dispase (Gibco Thermo Fisher Scientific) and incubated at 37 ​°C, 5% CO_2_, and 95% humidity overnight. Digested tissue was centrifuged for 5 ​min at 365 ​g and the resulting cell pellet re-suspended in DMEM supplemented with 50 U/mL penicillin (Gibco BRL, Life Technologies, Paisley, UK), 50 ​mg/mL streptomycin (Gibco BRL, Life Technologies) and 10% fetal calf serum (Gibco BRL, Life Technologies) and seeded in a 75 ​cm^2^ culture flask (Falcon, Corning Inc) and kept in an incubator at 37 ​°C, 5% CO_2_, and 95% humidity. For testing effects of intact dressings on proliferation, cells were seeded in 12-well culture plates at a density of 10,000 ​cells/well, allowed to attach overnight, covered with 10 ​mm diameter dressings (n ​= ​3), and cultured for 72 ​h. Every 24 ​h, cells were detached using 0.02% versene and 0.1% trypsin, and counted using an EVE automated cell counter (NanoEntek, Seoul, South Korea). To assess effects on migration, cells were expanded in 12-well culture plates until confluent, after which mitomycin was added to prevent proliferation and a scratch through the center of the well was produced using a p200 pipette tip. Cells were covered with 10 ​mm diameter dressings (n ​= ​3) and photographed every 24 ​h using an IX51 phase contrast microscope (Olympus, Solna, Sweden). Images were analyzed for repopulation of the denuded area using ImageJ software (National Institutes of Health, USA). Additionally, proliferation was analyzed using a LiveCyte II kinetic cytometer (Phase Focus, Sheffield, UK). BC-MSN-BTB dressings were prepared according to the protocol described above, and incubated in keratinocyte medium (keratinocyte serum-free medium (Gibco Thermo Fisher Scientific) supplemented with 25 ​μg/mL bovine pituitary extract, 1 ​ng/mL epidermal growth factor, 50 U/mL penicillin and 50 ​mg/mL streptomycin (PEST)) for 24 ​h in 37 ​°C. The obtained solution was serially diluted in keratinocyte medium in concentration 100 %–0% and dispensed on sub confluent keratinocytes. Cells were detached by 0.02% trypsin and 0.01% versine, counted using an EVE automated cell counter (NanoEntek), seeded at a concentration of 4000 ​cells/well in a 96-well plate (Flat bottom with Low Evaporation Lid, Tissue Culture Treated, Corning Incorporate Costar, Glendale, Arizona, USA) and incubated with 150 ​μL keratinocyte medium. The plate was cultured for 48 ​h prior to the beginning of the test when medium was substituted with release solution from the BC and BC composites (n ​= ​8). The experiment was carried on for 72 ​h, and images were acquired every 55 ​min (10× magnification, 1000× 1000 ​μm scan area). Data were processed using the Cell Analysis Toolbox software (Phase Focus, Sheffield, UK). All data from the cytocompatibility analyses were exported to Prism 8.0 (Graphpad, LaJolla, CA, US) for further analyses and generation of graphs. Statistical analysis was performed through non-parametric Kruskal–Wallis test completed with Dunn's multiple comparison post hoc test. For proliferation, data were processed in MATLAB R2019a (The MathWorks Inc., Natick, Massachusetts, United States) and noise was attenuated with Savigny-Golay filter. For migration, data were expressed in percent remaining of original scratch area at start of experiment.

*Porcine In Vivo Infected Wound Model:* Experiments involving the use of animals were performed under approval from the Regional Animal Ethics Committee (ID 1418), adhered to the guidelines postulated by Linköping University and were supervised by a veterinarian. All procedures were performed under general anesthesia, induced by intramuscular (IM) injection of 10 ​μg/kg dexmedetomidin (Dexdomitor; Orion Pharma, Danderyd, Sweden) and 3 ​μg/kg of tiletamin and zolazepam (Zoletil; Virbac, Kolding, Denmark). The animal (n ​= ​1) was intubated with an endotracheal tube connected to an automatic ventilator. General anesthesia and analgesia were maintained with intravenous infusion of 3–7.5 ​mg/kg pentobarbital sodium (Pentobarbitalnatrium vet.; APL, Kungens Kurva, Sweden) in combination with 0.5–0.75 ​μg/kg fentanyl (Leptanal, Janssen, Solna, Sweden). Vital parameters were monitored by pulse oximetry, capnography and rectal thermometer. Signs of postoperative pain were treated with IM administration of 50–75 ​μg fentanyl and 40 ​mg meloxicam (Loxicom; N-Vet AB, Uppsala, Sweden). Several circular full-thickness wounds with a diameter of 2 ​cm were created on the dorsum, of which two were used for testing the sensor. One of the wounds was inoculated with 2 ​× ​10^6^ colony forming units (CFU) of *Staphylococcus aureus* (ATCC 29213, Manassas, VA, USA). The remaining wound served as non-infected control. Two days later, the animal was anesthetized according to the above-described procedure and dressings were placed in direct contact with the wounds. Following the experiments, still under anesthesia, the animal was euthanized by intravenous injection of 400 ​mg/kg pentobarbital sodium (Pentobarbitalnatrium vet.; APL, Kungens Kurva, Sweden). Additionally, wound tissue was excised, fixed in 4% neutral buffered paraformaldehyde for 12 ​h, dehydrated through an ethanol–xylene series and embedded in paraffin. Using a microtome (RM2255, Leica Biosystems, Wetzlar, Germany), 6 ​μm sections of samples were mounted on slides and stained using Hematoxylin and Eosin (Histolab Products AB, Gothenburg, Sweden) according to manufacturer's instructions. Samples were visualized using an BX41 microscope (Olympus, Stockholm, Sweden) and images captured with a DP70 CCD camera (Olympus).

## 3 Results and discussion

### 3.1 BC-MSN nanocomposite materials

**Fig. 2:**
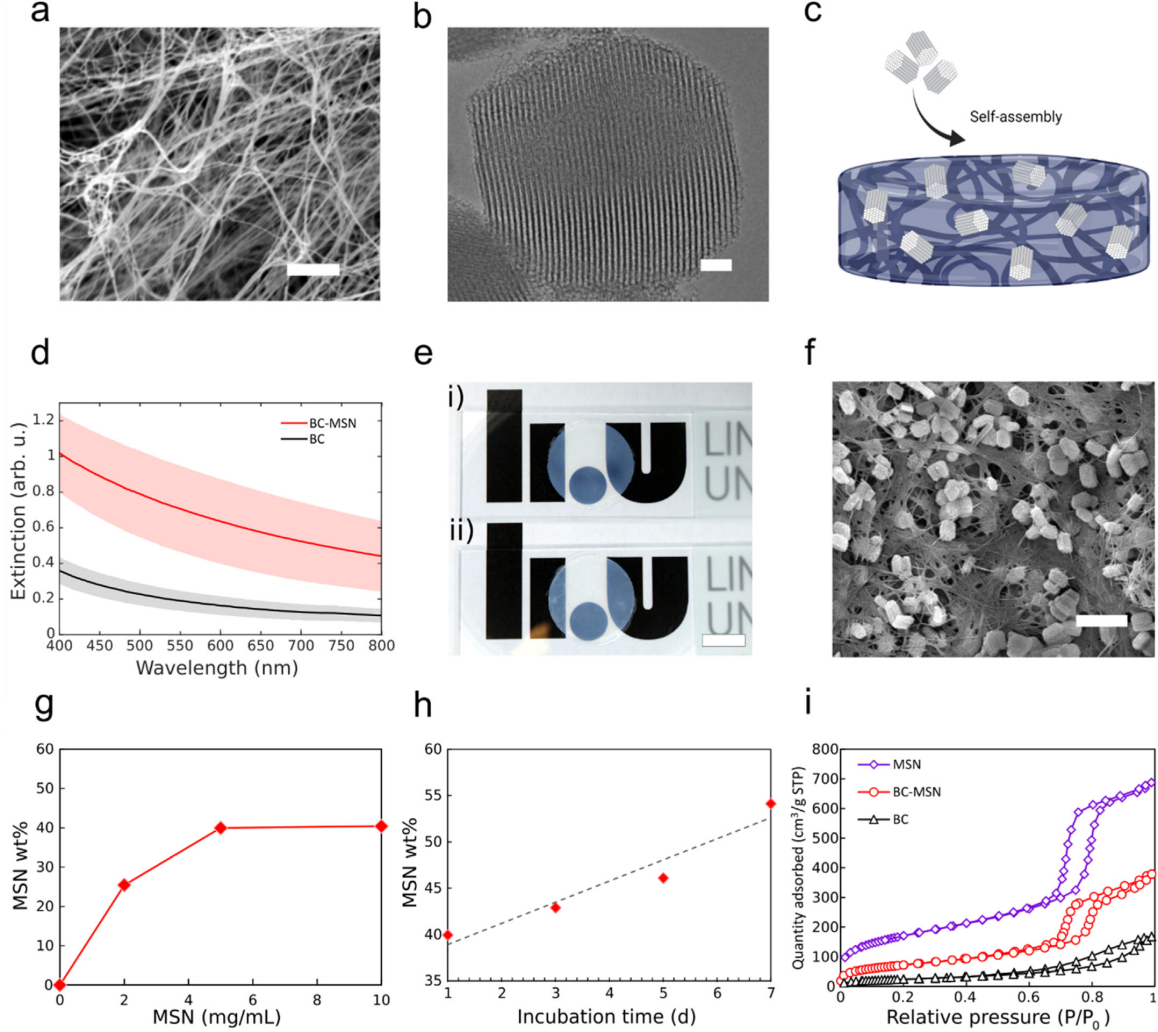
a) SEM micrograph of BC, scale bar: 2 ​μm ​b) ​TEM micrograph of MSN (SBA-15), scale bar: 50 ​nm. c) Schematic representation of MSN self-assembly process in BC. d) UV–vis spectra of BC and BC-MSN (5 ​mg/mL and 5 days incubation time), n ​> ​3. Shaded areas show standard deviations. e) Photographs of circular (ø 20 ​mm) BC (i) and BC-MSN (ii) dressings, scale bar: 1 ​cm. f) SEM micrograph of BC-MSN (5 ​mg/mL and 5 days incubation time), scale bar: 1 ​μm. g) Dry weight % of MSN in the BC dressings after incubation for 1 day at different MSN concentrations. h) Dry weight % of MSN in the BC dressings after incubation with BC for different times. i) Nitrogen physisorption isotherms of MSN, BC-MSN (5 ​mg/mL and 5 days incubation time) and BC.

BC is a translucent hydrogel consisting of a network of cellulose fibrils roughly 40–70 ​nm in width and up to several micrometers in length (Fig. 2a). The nanofibrillar networks form a highly hydrated and porous matrix. BC is chemically stable, and functionalization requires oxidation that, in addition to being laborious, can have a negative impact on the material properties [58]. To turn BC wound dressings into a pH-responsive material without the need to chemically functionalize the nanocellulose we explored the possibility of incorporating MSNs as a carrier of BTB (Fig. 2b and Fig. S1). Despite the negative zeta potentials of both BC [59,60] and MSNs [61] at neutral pH, we discovered that when BC was immersed in aqueous suspensions of MSNs with a defined ionic strength, the MSNs readily adsorbed to the BC (Fig. 2c and d). The accumulation of MSNs resulted in an increased scattering of light in the visible wavelength range compared to the native BC (Fig. 2d). However, these differences were small enough to not significantly affect the apparent transparency of the dressings (Fig. 2e), which facilitates the implementation of the colorimetric pH sensing strategy. The adsorption of MSNs to BC was further confirmed by scanning electron microscopy (SEM) (Fig. 2f and Fig. S2) and X-ray microtomography (XRT) (Fig. S3). The SEM micrographs show that large quantities of MSNs had diffused into the BC and were trapped in the porous network, apparently in close contact with the BC fibrils. The extensive MSN adsorption is most likely a result of short-range van der Waals interactions, as we have previously confirmed for other types of NPs [46]. This process requires that the particles are colloidally stable at sufficiently high ionic strength to compress the dielectric double layer to allow for close physical contact between the nanocellulose fibrils and the MSNs. Due to presence of residual salt from the synthesis, no additional increase in ionic strength was needed to promote adsorption. Increasing the ionic strength further did not significantly improve the adsorption rate (Fig. S4). Increasing the concentration of MSNs from 2 ​mg/mL to 5 ​mg/mL led to a 1.6-fold increase in MSN adsorption for incubation times of 1 day (Fig. 2g), as indicated by thermogravimetric analysis (TGA). Maintaining the concentration at 5 ​mg/mL, while increasing the incubation time from 1 day to 5 days resulted in increased accumulation of MSNs as observed by XRT scans (Fig. S3). When subjected to a suspension of MSNs (5 ​mg/mL) for 24 ​h, the dry weight of the BC dressings increased by 40%. After the first 24 ​h, the relative concentration of MSNs in the BC increased linearly with time, reaching about 55 ​wt % after 7 days of incubation (Fig. 2g,h and Fig. S5). The strong interactions between MSN and BC ensured a high retention of MSN nanoparticles and a negligible amount of the nanoparticles were lost from the dressing after 3 days of shaking in PBS (Fig. S6).

The presence of the MSNs further led to a dramatic increase in specific surface area. Due to the nanofibrillar network, BC alone has a relatively large specific surface area corresponding to about 88 ​m^2^/g as determined from nitrogen physisorption isotherms (Fig. 2i). The MSNs on the other hand, have a specific surface area of 600–700 ​m^2^/g. The BC-MSNs consequently display a large increase in effective surface area as compared to the BC alone, reaching 265 ​m^2^/g when incubated with a suspension of 5 ​mg/mL for 5 days. Increasing the concentration to 10 ​mg/mL and incubation time to 18 days lead to an additional 2.3-fold increase in effective surface area to 469 ​m^2^/g (Fig. S7), clearly showing the unique possibility to create hydrated nanocomposites with extremely high surface areas.

## 4 Fabrication pH-Responsive wound dressings

**Fig. 3:**
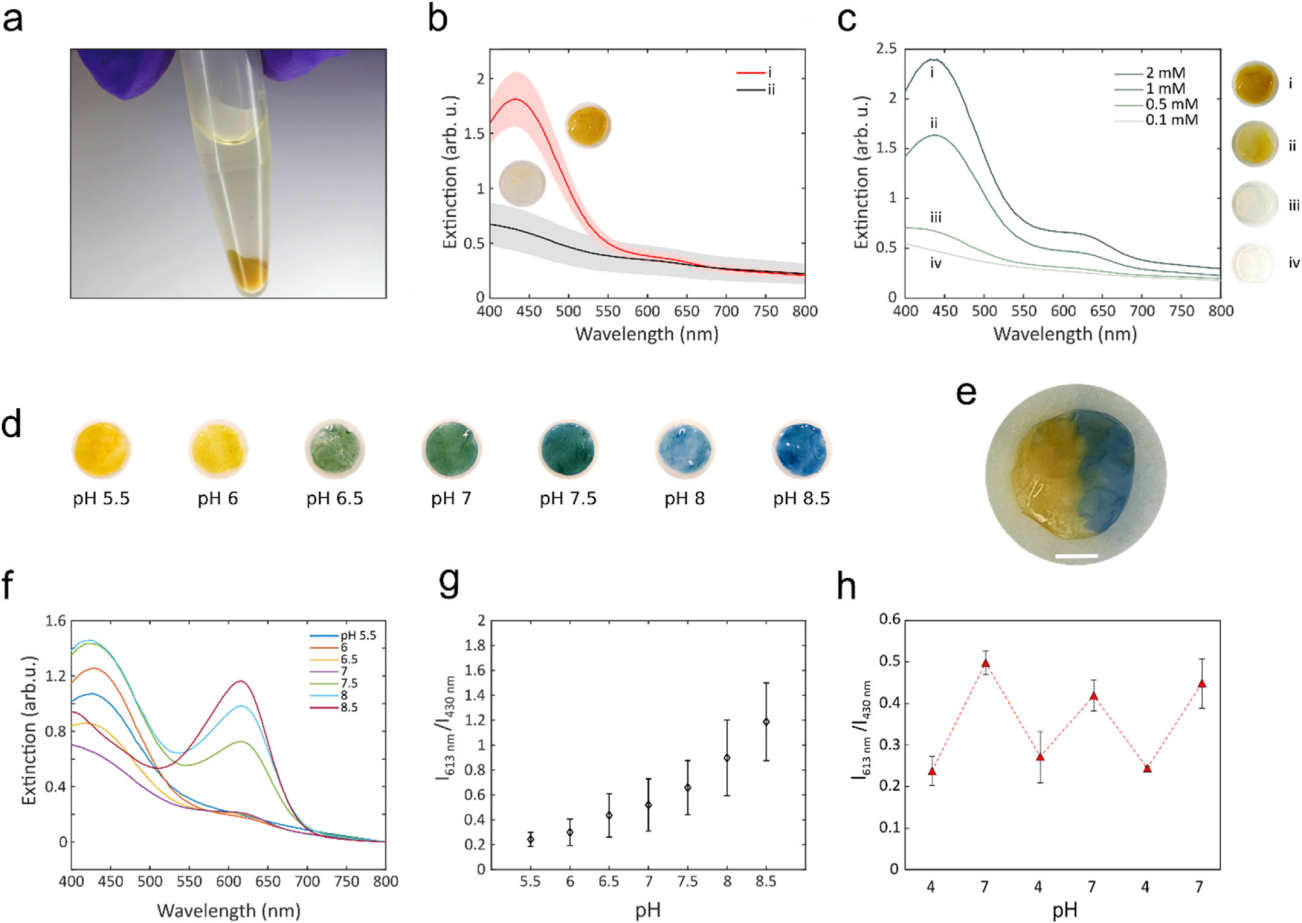
BTB-loading and pH-response of the nanocomposite wound dressings. a) Photograph of MSNs loaded with BTB and functionalized with PEI. b) pH@BC synthesis by i) BC-MSN incubated in BTB with subsequent functionalization of PEI, and ii) BTB and PEI pre-functionalized MSN loaded onto BC, n ​= ​6, shaded areas show standard deviation. c) Effect of varying BTB concentration on sensor color intensity. d) Photographs of pH@BC at different pH values ranging from pH 5.5 to 8.5. e) Spatial pH sensing with a pH-responsive dressing subject to: left pH ​< ​6 and right pH ​> ​8, scale bar: 2 ​mm. f) Extinction spectra of pH@BC at different pH measured between 400 and 800 ​nm. g) Ratio between the two BTB peaks (∼430 and ∼613 ​nm) as a function of pH between pH 5.5 and 8.5, n = 3. h) BTB peak ratio for a sensor repeatedly exposed to buffers with pH 4 and 7, n ​= ​2. Error bars show standard deviations. (For interpretation of the references to colour in this figure legend, the reader is referred to the Web version of this article.)

To create pH-responsive wound dressings (pH@BC), we exploited the capacity of MSNs to adsorb and retain a low molecular weight pH responsive dye (BTB). BTB was found to adsorb readily in MSNs, both in suspension (Fig. 3a) and when bound to BC (Fig. 3b). After loading the MSNs with BTB, the pores were capped by the polyelectrolyte polyethyleneimine (PEI) to prevent release of the adsorbed dye. Without capping, the BTB rapidly dissociated from the MSNs whereas MSNs capped with PEI retained the majority of the loaded BTB without any substantial loss in color intensity over time (Fig. S8). In absence of MSNs, the color of the dressings was very weak, indicating poor binding of BTB to the nanocellulose and demonstrating the necessity of using MSNs for efficient immobilization and long-term retention of BTB, (Fig. S9). Storage of PEI capped BTB-loaded BC-MSN dressings kept in water for up to 14 days did not result in any decrease in color intensity (Fig. S10). The color intensity of the final pH@BC materials could be tuned by the BTB concentration in the incubation solution. A higher BTB concentration resulted in a more intense color of the pH@BC dressings but did not alter the pH sensing properties of the encapsulated dye (Fig. 3c). Additionally, we investigated a second synthesis route by pre-loading the MSNs particles with BTB, followed by capping with PEI, after which they were adsorbed to BC (Fig. 3b). Although the BTB could be efficiently retained in the MSNs, the adsorption of the MSNs to BC was less efficient, most likely due to the PEI capping reducing the van der Waals interactions between BC and MSNs [46].

We further investigated the pH-responsiveness of the final composite material by immersing the pH@BC dressings in buffers with different pH. We noticed a rapid (seconds) color transition from yellow at pH 5.5 to green at pH 7 and further to blue at pH 8 and above (Fig. 3d). Additionally, the dressings could pick up differences in pH with spatial resolution (Fig. 3e). UV–vis spectra of the dressings in Fig. 3d recorded in transmission mode showed a distinct spectral change as pH was increased (Fig. 3f). The spectra showed a similar trend as the colorimetric response of BTB in solution (Fig. S11), with some minor differences caused by light scattering from both the MSNs and BC. By using the ratio of the extinction peaks at 613 ​nm and 430 ​nm, it was possible to reduce any effects caused by differences in BTB concentrations and particle distributions within and between dressings (Fig. 3g). Moreover, the pH-induced color changes were reversible and cycling between pH 4 and 7 resulted in reproducible spectral changes (Fig. 3h). The pH@BC dressings are hence able to monitor dynamic changes in wound pH, which could be highly relevant for investigating, for example, effects on interventions aiming at reducing the bacterial load.

### 4.1 Wound dressing characteristics

**Fig. 4:**
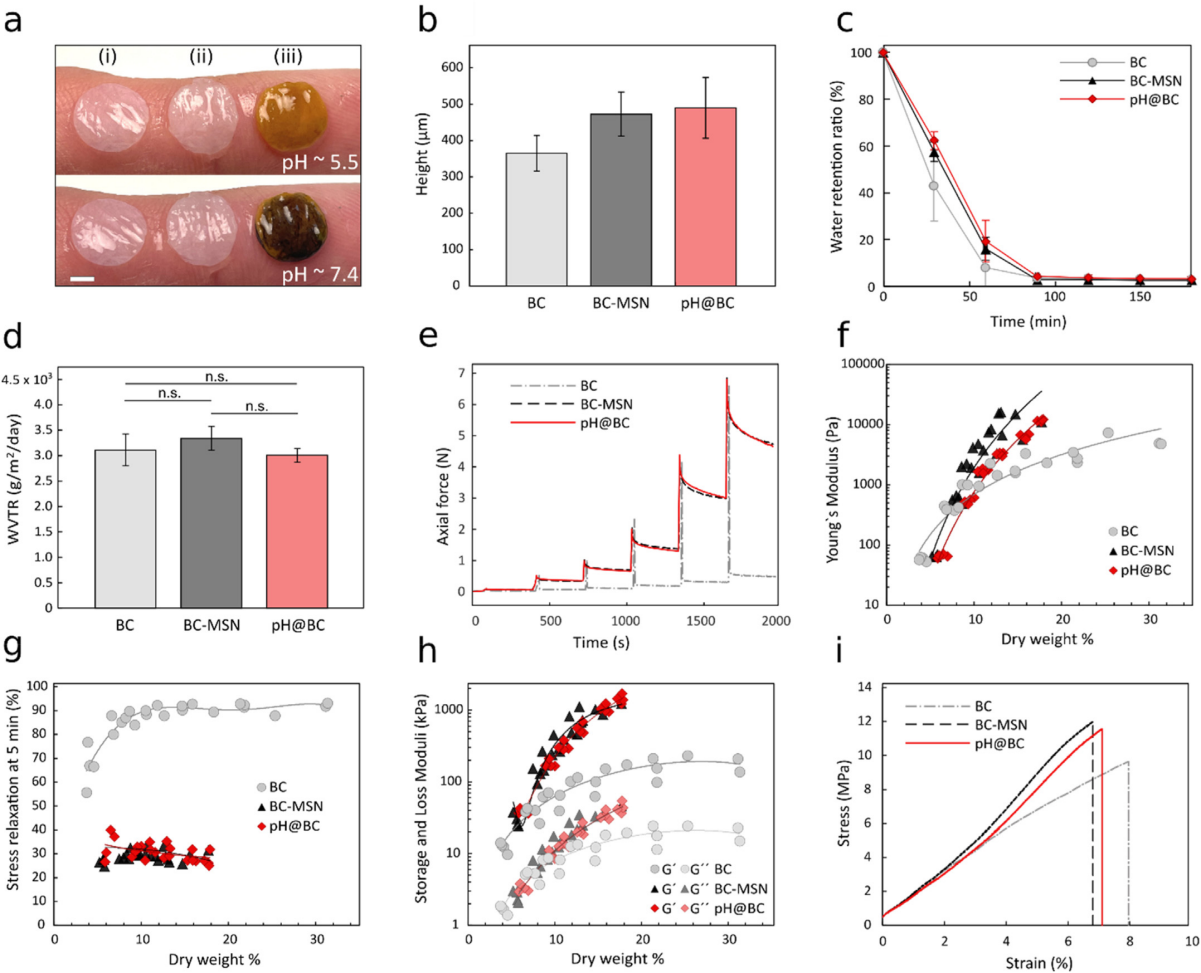
Physical and mechanical properties of BC-MSN composite wound dressings. a) Conformability of 6 ​mm dressings of (i) BC, (ii) BC-MSN and (iii) pH@BC on untreated skin (pH ​∼ ​5.5) and skin subject to a pH 7.4 buffer (scale bar: 2 ​mm). b) Thickness of BC dressings, BC-MSN and pH@BC, n ​≥ ​6. Error bars show standard deviations. c) Water retention ratio (WRR), n ​≥ ​5. Error bars show standard deviation. d) WVTR of BC, BC-MSN and pH@BC dressings, n ​= ​5. Error bars show standard deviation. Significance was tested using one way ANOVA, p ​< ​0.01. e–h) Rheological compression-relaxation characterization of BC, BC-MSN and pH@BC dressings. e) Axial force as a function of time. f) Young's modulus as a function of dry weight %. g) Stress relaxation after 5 ​min from the onset of stress relaxation step as a function of dry weight %. h) Storage and loss moduli (G′, G″) evaluated under oscillatory shear during relaxation as a function of dry weight %. i) Representative stress and strain curves from tensile testing of BC, BC-MSN, and pH@BC dressings.

Native BC wound dressings conform very well to both intact skin and wounds, resulting in a close contact between the material and the tissue. This close contact between the wound and the dressing reduces pain and promotes healing [62]. The functionalization of the BC dressings with MSNs resulted in a material that retained the conformability of BC, while presenting a slightly more open and hydrated structure (Fig. 4a). The incorporation of MSNs increased the thickness of the dressings by about 30% with respect to unfunctionalized BC (Fig. 4b), from 366 ​± ​49 ​μm for native BC to 473 ​± ​61 ​μm for the composite material. Due to the increase in volume and meso-scale porosity, the composite material showed an increased fluid retention capacity (Fig. 4c).

An optimal wound dressing should have evaporative water loss values that can balance wound bed dehydration and exudate accumulation with an ideal WVTR between 2000 and 2500 ​g/m^2^/day [63], which is close to the values observed for native BC dressings (Fig. 4d). Introducing MSNs did not significantly alter the WVTR, nor did BTB loading and PEI functionalization. The pH@BC dressings thus retain the close to ideal WVTR of native BC dressings.

Both the handling and performance of the dressings are highly dependent on the mechanical properties of the materials. To assess the impact of the presence of MSNs in the BC network, we first measured the mechanical properties of the dressings in the out-of-plane direction as a function of the dry content of the materials. When compressed, the BC network shows very little deformation in the radial direction [64], which makes it possible to estimate the sample volume from the geometry gap in the rheometer during the measurement. Subsequently, the changes in relative dry content can be derived from the gap height and the initial dry weight. Water content measurements demonstrated that BC fibrils accounted for 2.85 ​wt % and MSNs for 1.95 ​wt %.

To perform a quantitative comparison of the mechanical properties of the three materials, we related the mechanical properties to the relative dry weight of the dressings. During axial compression, the material response was dominated by flow of free water out of the BC network in the radial direction. The water flow is highly influenced by the BC network porosity. To study the porosity effect on the water flow, the compression was followed by a stress relaxation step during which the material was allowed to relax under steady compressive conditions. We evaluated the material stress relaxation response as the drop in normal force over a period of 5 ​min (Fig. 4e–h). The compressive stiffness of the dressings was evaluated in terms of Young's modulus. The addition of MSNs increased the stiffness of the dressings through the formation of a load-bearing network, which allowed the materials to reach higher compressive stresses upon moderate compression strains (Fig. 4f). The MSNs also influenced the stress relaxation of the dressings. Native BC showed high stress decay rates with over 90% decay within 3 ​s for compression forces ≥ 1 ​N and full relaxation (static state) within 1 ​min for all the measured compressive force levels. The MSN-functionalized BC showed incomplete relaxation and substantial changes in the relaxation profile (Fig. 4e). The MSN-containing dressings demonstrated a low stress decay rate, which was more pronounced at high compression levels (Fig. S12). In addition, longer stress relaxation times were observed where relaxation extended to 45% after 1 ​h, following ∼12 ​kPa compression, without reaching static state (Fig. S13). These results indicate that the MSN-functionalized dressings have better ability to withstand compressive forces, which is of clinical relevance, especially when applied in joint regions where non-intentional compressions can occur. This effect is partly caused by the MSNs interacting with multiple BC fibrils, stiffening the material. Moreover, the increased tortuosity of the hydrogel network following the addition of the MSNs hinders water flow. As expected, no additional effect on the stress relaxation behavior were seen after loading the MSNs with BTB and capping with PEI (Fig. 4g).

Both BC and BC-MSN composites form viscoelastic networks with a storage modulus (G′) higher than the loss modulus (G″). The presence of the MSNs increased stiffness of the materials compared to native BC at the same compression level. Furthermore, the increase in G′ over time, while undergoing small amplitude oscillation strains, suggests establishment of additional strong interactions between MSNs and BC fibrils caused by the mechanical rearrangements in the composites (Fig. 4h). Despite the differences in mechanical properties upon compression, tensile strength measurements only showed minor differences between native BC and the different composites (Fig. 4i). We observed a small increase in tensile strength in the presence of MSNs compared to native BC, further indicating that the MSNs interact with multiple nanocellulose fibrils resulting in a reinforcement of the materials.

### 4.2 Cytocompatibility and *in vivo* performance

**Fig. 5:**
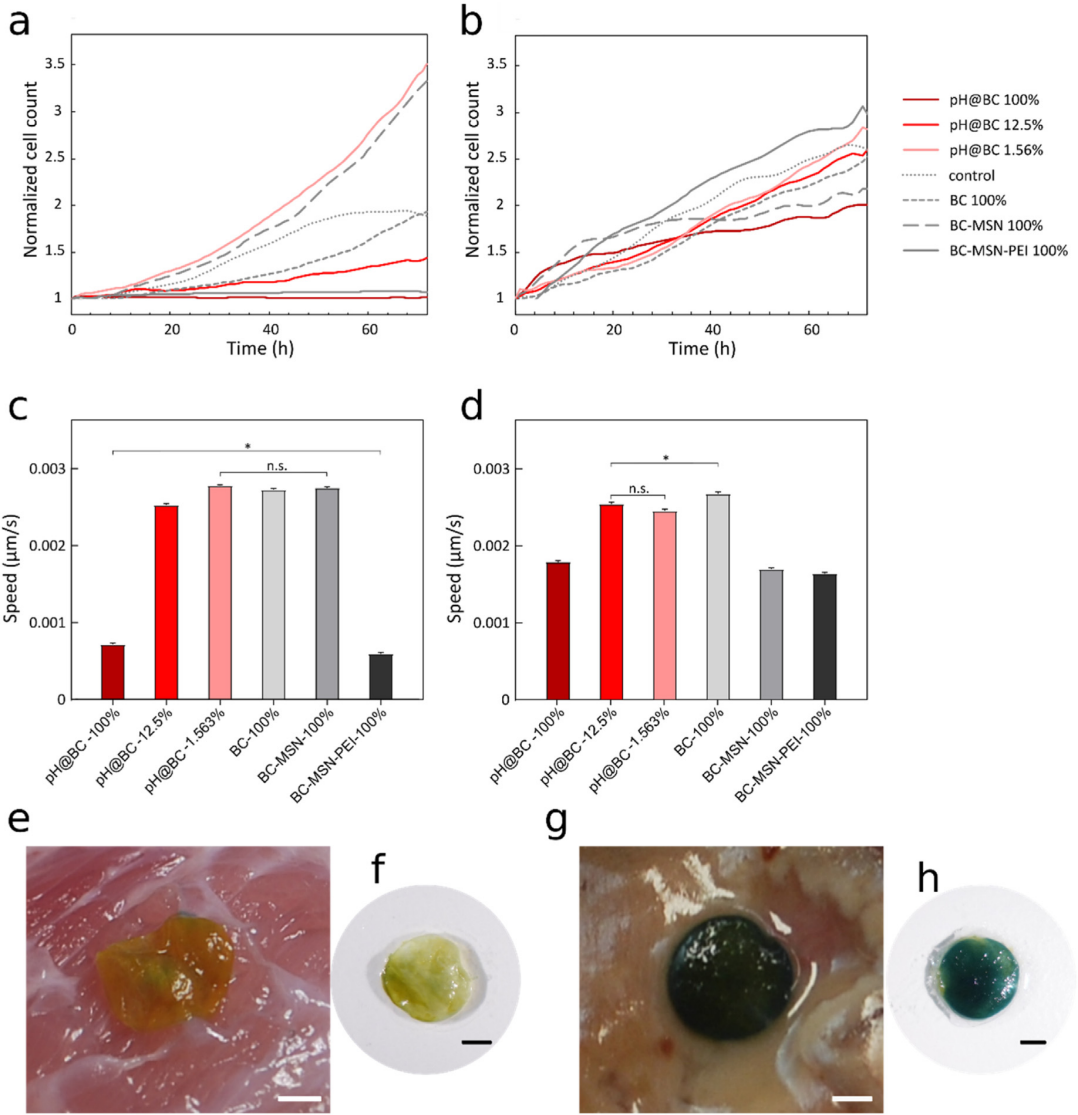
Biocompatibility testing of the pH-responsive wound dressings. a, c) Keratinocyte and b, d) fibroblast normalized cell count and migration rate over 72 ​h when cultured in extracts from pH@BC. Results displayed as mean and standard error of the mean, ∗P ​< ​0.1; P ​< ​0.0001 whereas not indicated (n ​= ​8). Photographs of pH-responsive wound dressings applied on (e) non-infected wound and (g) a wound infected with *Staphylococcus aureus*, scale bar: 2 ​mm. Photographs were recorded 1 ​min after application of the dressings. f, h) show dressings (e) and (g), respectively, after being recovered from the wound and rinsed in water, scale bar: 2 ​mm.

The cytocompatibility of the dressings was investigated according to ISO 10993–5:2009 by first exposing cultured human primary keratinocytes and fibroblasts to leachables from the BC nanocomposites, obtained by incubating the dressings in cell culture medium for 24 ​h at 37 ​°C. The cells were cultured for 72 ​h and cell proliferation and migration were measured continuously. For the lower concentrations of leachables, there was no decrease in proliferation for neither keratinocytes nor fibroblasts compared to the controls (Fig. 5a and b and Fig. S14). For the highest concentrations of leachables, cell proliferation was reduced for keratinocytes but not for fibroblasts. A decrease in keratinocyte migration rate was seen at high concentrations of leachables whereas cells cultured at lower concentrations were largely unaffected compared to the controls (Fig. 5c, Fig. S15a and Fig. S16). No substantial differences in fibroblast migration were seen for any of the conditions (Fig. 5d, Fig. S15b and Fig. S17). Moreover, no differences in cell response between BC-MSN-PEI and pH@BC were observed. Both fibroblasts and keratinocytes thus tolerate the components leaching from the dressings. This is in line with previous observations that BTB does not influence cell viability at concentration <1 ​mg/mL [65,66]. To further explore cytocompatibility of the dressings, they were also placed directly on top of the cultured cells. A decrease in cell proliferation but not in cell number was seen for all conditions, including native BC, compared to the non-covered cells for both fibroblasts (Fig. S18a) and keratinocytes (Fig. S18b). Cell migration of covered cells were assessed using a scratch assay. All conditions, including native BC, showed a decrease in migration rate compared to the non-covered cells for both fibroblasts (Fig. S18c) and keratinocytes (Fig. S18d). Since cells also covered by native BC showed reduced proliferation and migration, the effects are likely primarily due to the restriction in gas and nutrient diffusion over the dressings. In a wound, however, nutrient and oxygen transport primarily occurs from the wound bed and cells will likely not be affected to the same extent when covered by the dressings.

In the *in vivo* situation, the wound environment is highly complex, and the dressings will be in direct contact with tissues and wound exudate [67], and in the case of infected wounds also bacteria and biofilms. To study the *in vivo* performance of the pH-responsive wound dressings we used a porcine wound model due to demonstrated similarities in cellular composition, morphology, and immunological properties to human skin [68]. The pH-responsive dressings were applied to both a non-infected wound and a wound infected with *Staphylococcus aureus* (Fig. S19). The size of the dressings was intentionally kept smaller than the wound during these experiments to ensure close contact between the dressings and the tissue. In the non-infected wound, the dressing showed a yellow-greenish color indicative of physiological pH (Fig. 5e and f). In contrast, the dressings applied on the infected wound showed a rapid (<1 ​min) change in color from yellow to deep blue, indicating an alkaline environment with a pH ∼8 (Fig. 5g and h). The color of the dressing in the infected wound showed a distinct contrast to the color of the tissue, allowing for a clear sensor readout. The experiments thus indicate that the color change of the dressings occur as rapidly *in vivo* as when subjected to pure buffers and confirmed that sensor readout is possible also directly in the wound.

## 5 Conclusions

Nanocomposite wound dressings with integrated real-time pH sensing properties were developed using a self-assembly-based fabrication strategy for integrating MSNs in BC. The MSNs were loaded with a pH-responsive dye (BTB) and capped with PEI to prevent release of the dye. The BC-based nanocomposites retained excellent wound dressing material characteristics, similar to native BC, including good conformability allowing for close contact between the dressing and the wound bed. The pH@BC dressings showed appropriate exudate holding capacity and water vapor transmission rate, which are important for preservation of a moist wound environment and wound re-epithelialization. The self-assembly fabrication process further allowed for tuning of the material characteristics, including mechanical properties and effective surface area. An increase in effective surface area from 88 ​m^2^/g of native BC to 469 ​m^2^/g after functionalization with MSNs was obtained. Without MSNs, the dressings could not retain the BTB and consequently lost the pH sensing capabilities. The pH@BC wound dressings were tolerated by cultured human primary keratinocytes and fibroblasts, the main cell types of human skin. The large surface area and mesoporous structure of the BC-MSN composites allowed for efficient loading and retention of pH-responsive dyes, enabling real-time wound pH monitoring with spatiotemporal resolution and clear readout both *in vitro* and *in vivo* in an infected porcine wound model. The possibility to continuously monitor wound pH without having to remove the dressing can shed new light on the effect of wound pH on healing and enable early detection of wound infections, allowing for improved diagnosis and treatment that can halt progression and development of non-healing wounds. Moreover, naked-eye sensor readout can facilitate cost-effective and streamlined wound care, potentially reducing needs for excessive treatment with antibiotics. The possibilities to create well-defined composites of two high-surface-area biomaterials can further enable development of a wide range of devices for therapeutic and diagnostic applications for advanced wound care applications.

## CRediT author statement

**Olof Eskilson:** Investigation, Formal analysis, Validation, Writing original draft. **Elisa Zattarin:** Investigation, Formal analysis, Validation, Writing original draft. **Linn Berglund:** Investigation, Formal analysis. **Kristiina Oksman:** Supervision, Funding acquisition, Resources. **Kristina Hanna:** Investigation, Formal analysis. **Jonathan Rakar:** Investigation, Formal analysis. **Petter Sivlér:** Conceptualization, Resources. **Mårten Skog:** Conceptualization, Resources. **Ivana Rinklake:** Investigation, Formal analysis. **Rozalin Shamasha:** Investigation, Formal analysis. **Zeljana Sotra:** Investigation, Formal analysis. **Annika Starkenberg:** Investigation, Formal analysis. **Magnus Odén:** Resources, Supervision, Funding acquisition. **Emanuel Wiman:** Investigation, Formal analysis. **Hazem Khalaf:** Investigation, Formal analysis, Supervision. **Torbjörn Bengtsson:** Resources, Supervision, Funding acquisition. **Johan P. E. Junker:** Formal analysis, Writing original draft, Supervision, Funding acquisition, Writing - Review & Editing. **Robert Selegård:** Conceptualization, Supervision, Investigation, Formal analysis, Writing - Review & Editing. **Emma M. Björk:** Conceptualization, Investigation, Formal analysis. **Daniel Aili:** Conceptualization, Formal analysis, Writing original draft, Writing - Review & Editing, Supervision, Funding acquisition.

## Declaration of competing interest

The authors declare that they have no known competing financial interests or personal relationships that could have appeared to influence the work reported in this paper.

## Data Availability

Data will be made available on request.

## Appendix A Supplementary data

Additional SEM micrographs of MSNs and BC-MSN nanocomposites, XRT data and reconstruction in 2D and 3D of BC-MSN, TGA of BC-MSN loaded in suspensions of different concentrations of NaCl, additional TGA data of BC and BC-MSN, BET surface area plot of BC and BC-MSN, data on effects of PEI capping on BTB retention and data on BTB release, stability of BT-MSN, UV-vis spectra of BTB, stress relaxation data, cell proliferation and motility data of keratinocytes and fibroblasts cultured in pH@BC leachables, cell proliferation data of keratinocytes and fibroblasts covered by pH@BC membranes, histology of infected burn wound.

## References

[1] Leaper D., Assadian O., Edmiston C.E. (2015). Approach to chronic wound infections. Br. J. Dermatol..

[2] Haesler E., Ousey K., Lecturer H.S., Associate H., Prevention I. (2018). Clinical practice Evolution of the wound infection continuum. Wounds Int..

[3] Singer M., Deutschman C.S., Seymour C., Shankar-Hari M., Annane D., Bauer M., Bellomo R., Bernard G.R., Chiche J.D., Coopersmith C.M., Hotchkiss R.S., Levy M.M., Marshall J.C., Martin G.S., Opal S.M., Rubenfeld G.D., Der Poll T., Vincent J.L., Angus D.C. (2016). The third international consensus definitions for sepsis and septic shock (sepsis-3). JAMA, J. Am. Med. Assoc..

[4] Huang C.Y., Daniels R., Lembo A., Hartog C., O'Brien J., Heymann T., Reinhart K., Nguyen H.B., Azevedo L., Finfer S., Fleischmann C., Foreman T., Kissoon N., Machado F., Martens C., Possagnoli I., Hanke R., Vroomen-Durning M. (2019). Life after sepsis: an international survey of survivors to understand the post-sepsis syndrome. Int. J. Qual. Health Care.

[5] Werdin F., Tenenhaus M., Rennekampff H.O. (2008). Chronic wound care. Lancet.

[6] Kyaw B.M., Järbrink K., Martinengo L., Car J., Harding K., Schmidtchen A. (2018). Need for improved definition of “chronic wounds” in clinical studies. Acta Derm. Venereol..

[7] Sen C.K., Gordillo G.M., Roy S., Kirsner R., Lambert L., Hunt T.K., Gottrup F., Gurtner G.C., Longaker M.T. (2009). Human skin wounds: a major snoballing threat to public Health and economy. Wound Repair Regen..

[8] Olsson M., Järbrink K., Divakar U., Bajpai R., Upton Z., Schmidtchen A., Car J. (2019). The humanistic and economic burden of chronic wounds: a systematic review. Wound Repair Regen..

[9] Järbrink K., Ni G., Sönnergren H., Schmidtchen A., Pang C., Bajpai R., Car J. (2017). The humanistic and economic burden of chronic wounds: a protocol for a systematic review. Syst. Rev..

[10] Sen C.K. (2019). Human wounds and its burden: an updated compendium of estimates. Adv. Wound Care.

[11] Nussbaum S.R., Carter M.J., Fife C.E., DaVanzo J., Haught R., Nusgart M., Cartwright D. (2018). An economic evaluation of the impact, cost, and medicare policy implications of chronic nonhealing wounds. Value Health.

[12] Friedman N.D., Temkin E., Carmeli Y. (2016). The negative impact of antibiotic resistance. Clin. Microbiol. Infect..

[13] Martin P., Nunan R. (2015). Cellular and molecular mechanisms of repair in acute and chronic wound healing. Br. J. Dermatol..

[14] Gethin G., Cowman S., Kolbach D.N. (2017). Debridement for venous leg ulcers. Cochrane Database Syst. Rev..

[15] Evelhoch S.R. (2020). Biofilm and chronic nonhealing wound infections. Surg. Clin. NA.

[16] Li S., Renick P., Senkowsky J., Nair A., Tang L. (2021). Diagnostics for wound infections. Adv. Wound Care.

[17] Lazarus G.S., Cooper D.M., Knighton D.R., Margolis D.J., Percoraro R.E., Rodeheaver G., Robson M.C. (1994). Definitions and guidelines for assessment of wounds and evaluation of healing. Wound Repair Regen..

[18] Belushkin A., Yesilkoy F., González-López J.J., Ruiz-Rodríguez J.C., Ferrer R., Fàbrega A., Altug H. (2020). Rapid and digital detection of inflammatory biomarkers enabled by a novel portable nanoplasmonic imager. Small.

[19] Zhou C., Tang N., Zhang X., Fang Y., Jiang Y., Zhang H., Duan X. (2020). Simultaneously optimize the response speed and sensitivity of low dimension conductive polymers for epidermal temperature sensing applications. Front. Chem..

[20] Charaya H., La T.G., Rieger J., Chung H.J. (2019). Thermochromic and piezocapacitive flexible sensor array by combining composite elastomer dielectrics and transparent ionic hydrogel electrodes. Adv. Mater. Technol..

[21] Pan N., Qin J., Feng P., Li Z., Song B. (2019). Color-changing smart fibrous materials for naked eye real-time monitoring of wound pH. J. Mater. Chem. B.

[22] Rahimi R., Ochoa M., Parupudi T., Zhao X., Yazdi I.K., Dokmeci M.R., Tamayol A., Khademhosseini A., Ziaie B. (2016). A low-cost flexible pH sensor array for wound assessment. Sensor. Actuator. B Chem..

[23] Jankowska D.A., Bannwarth M.B., Schulenburg C., Faccio G., Maniura-Weber K., Rossi R.M., Scherer L., Richter M., Boesel L.F. (2017). Simultaneous detection of pH value and glucose concentrations for wound monitoring applications. Biosens. Bioelectron..

[24] Schneider L.A., Korber A., Grabbe S., Dissemond J. (2007). Influence of pH on wound-healing: a new perspective for wound-therapy?. Arch. Dermatol. Res..

[25] Wilson I.A.I., Henry M., Quill R.D., Byrne P.J. (1979). The pH of varicose ulcer surfaces and its relationship to healing. Vasa - J. Vasc. Dis..

[26] Tsukada K., Tokunaga K., Iwama T., Mishima Y. (1992). The pH changes of pressure ulcers related to the healing process of wounds. Wounds.

[27] Leveen H.H., Falk G., Borek B., Diaz C., Lynfield Y., Wynkoop B.J., Mabunda G.A., Rubricius J.L., Christoudias G.C. (1973). Chemical acidification of wounds. An adjuvant to healing and the unfavorable action of alkalinity and ammonia. Ann. Surg..

[28] Gethin G. (2007). The significance of surface pH in chronic wounds. Wounds U. K..

[29] Jones E.M., Cochrane C.A., Percival S.L. (2015). The effect of pH on the extracellular matrix and biofilms. Adv. Wound Care.

[30] Metcalf D.G., Haalboom M., Bowler P.G., Gamerith C., Sigl E., Heinzle A., Burnet M.W.M. (2019). Elevated wound fluid pH correlates with increased risk of wound infection. Wound Med..

[31] Ono S., Imai R., Ida Y., Shibata D., Komiya T., Matsumura H. (2015). Increased wound pH as an indicator of local wound infection in second degree burns. Burns.

[32] Bennison L.R., Miller C., Summers R., Minnis A., Sussman G., Mcguiness W. (2017). The pH of wounds during healing and infection: a descriptive literature review. J. Aust. Wound Manag. Assoc..

[33] Jiang L., Loo S.C.J. (2021). Intelligent nanoparticle-based dressings for bacterial wound infections. ACS Appl. Bio Mater..

[34] Liang Y., Xu H., Li Z., Zhangji A., Guo B. (2022). Bioinspired injectable self-healing hydrogel sealant with fault-tolerant and repeated thermo-responsive adhesion for sutureless post-wound-closure and wound healing. Nano-Micro Lett..

[35] Moradpoor H., Mohammadi H., Safaei M., Mozaffari H.R., Sharifi R., Gorji P., Sulong A.B., Muhamad N., Ebadi M. (2022). Recent advances on bacterial cellulose-based wound management: promises and challenges. Int. J. Polym. Sci..

[36] Liang Y., Li M., Yang Y., Qiao L., Xu H., Guo B. (2022). pH/Glucose dual responsive metformin release hydrogel dressings with adhesion and self-healing via dual-dynamic bonding for athletic diabetic foot wound healing. ACS Nano.

[37] Dong R., Guo B. (2021). Smart wound dressings for wound healing. Nano Today.

[38] Mirani B., Pagan E., Currie B., Siddiqui M.A., Hosseinzadeh R., Mostafalu P., Zhang Y.S., Ghahary A., Akbari M. (2017). An advanced multifunctional hydrogel-based dressing for wound monitoring and drug delivery. Adv. Healthc. Mater..

[39] Lee K.Y., Mooney D.J. (2012). Alginate: properties and biomedical applications. Prog. Polym. Sci..

[40] Aderibigbe B.A., Buyana B. (2018). Alginate in wound dressings. Pharmaceutics.

[41] Liu L., Li X., Nagao M., Elias A.L., Narain R., Chung H.-J. (2017). A pH-Indicating colorimetric tough hydrogel patch towards applications in a substrate for smart wound dressings. Polymers.

[42] Gamerith C., Luschnig D., Ortner A., Pietrzik N., Guse J.H., Burnet M., Haalboom M., van der Palen J., Heinzle A., Sigl E., Gübitz G.M. (2019). pH-responsive materials for optical monitoring of wound status. Sensor. Actuator. B Chem..

[43] Tamayol A., Akbari M., Zilberman Y., Comotto M., Lesha E., Serex L., Bagherifard S., Chen Y., Fu G., Ameri S.K., Sonkusale S., Khademhosseini A. (2016). Flexible pH-sensing hydrogel fibers for epidermal applications. Adv. Healthc. Mater..

[44] Karlsson M., Olofsson P., Steinvall I., Sjöberg F., Thorfinn J., Elmasry M. (2019). Three years' experience of a novel biosynthetic cellulose dressing in burns. Adv. Wound Care.

[45] delli Santi G., Borgognone A. (2019). The use of Epiprotect®, an advanced wound dressing, to heal paediatric patients with burns: a pilot study. Burns Open.

[46] Eskilson O., Lindström S.B., Sepulveda B., Shahjamali M.M., Güell-Grau P., Sivlér P., Skog M., Aronsson C., Björk E.M., Nyberg N., Khalaf H., Bengtsson T., James J., Ericson M.B., Martinsson E., Selegård R., Aili D. (2020). Self-assembly of mechanoplasmonic bacterial cellulose–metal nanoparticle composites. Adv. Funct. Mater..

[47] Wu P.H., Mäkie P., Odén M., Björk E.M. (2019). Growth and functionalization of particle-based mesoporous silica films and their usage in catalysis. Nanomaterials.

[48] Dindar M.H., Yaftian M.R., Rostamnia S. (2015). Potential of functionalized SBA-15 mesoporous materials for decontamination of water solutions from Cr(VI), As(V) and Hg(II) ions. J. Environ. Chem. Eng..

[49] Heidari A., Younesi H., Mehraban Z. (2009). Removal of Ni(II), Cd(II), and Pb(II) from a ternary aqueous solution by amino functionalized mesoporous and nano mesoporous silica. Chem. Eng. J..

[50] Ashley C.E., Carnes E.C., Phillips G.K., Padilla D., Durfee P.N., Brown P.A., Hanna T.N., Liu J., Phillips B., Carter M.B., Carroll N.J., Jiang X., Dunphy D.R., Willman C.L., Petsev D.N., Evans D.G., Parikh A.N., Chackerian B., Wharton W., Peabody D.S., Brinker C.J. (2011). The targeted delivery of multicomponent cargos to cancer cells by nanoporous particle-supported lipid bilayers. Nat. Mater..

[51] He Q., Shi J. (2011). Mesoporous silica nanoparticle based nano drug delivery systems: synthesis, controlled drug release and delivery, pharmacokinetics and biocompatibility. J. Mater. Chem..

[52] Rahikkala A., Pereira S.A.P., Figueiredo P., Passos M.L.C., Araújo A.R.T.S., Saraiva M.L.M.F.S., Santos H.A. (2018). Mesoporous silica nanoparticles for targeted and stimuli-responsive delivery of chemotherapeutics: a review. Adv. Biosyst..

[53] Chen Y., Chen H., Shi J. (2013). Vivo bio-safety evaluations and diagnostic/therapeutic applications of chemically designed mesoporous silica nanoparticles. Adv. Mater..

[54] Björk E.M., Atakan A., Wu P.H., Bari A., Pontremoli C., Zheng K., Giasafaki D., Iviglia G., Torre E., Cassinelli C., Morra M., Steriotis T., Charalambopoulou G., Boccaccini A.R., Fiorilli S., Vitale-Brovarone C., Robertsson F., Odén M. (2021). A shelf-life study of silica- and carbon-based mesoporous materials. J. Ind. Eng. Chem..

[55] Björk E.M., Söderlind F., Odén M. (2013). Tuning the shape of mesoporous silica particles by alterations in parameter space: from rods to platelets. Langmuir.

[56] Slowing I.I., Vivero-Escoto J.L., Wu C.W., Lin V.S.Y. (2008). Mesoporous silica nanoparticles as controlled release drug delivery and gene transfection carriers. Adv. Drug Deliv. Rev..

[57] Huh S., Wiench J.W., Yoo J.C., Pruski M., Lin V.S.Y. (2003). Organic functionalization and morphology control of mesoporous silicas via a Co-condensation synthesis method. Chem. Mater..

[58] Mohammadkazemi F., Khademibarangenani R., Koosha M. (2019). The effect of oxidation time and concentration on physicochemical, structural, and thermal properties of bacterial nano-cellulose. Polym. Sci..

[59] el Mrabate B., Udayakumar M., Csiszár E., Kristály F., Leskó M., Somlyai Sipos L., Schabikowski M., Németh Z. (2020). Development of bacterial cellulose–ZnO–MWCNT hybrid membranes: a study of structural and mechanical properties. R. Soc. Open Sci..

[60] Lee K.-Y., Quero F., Blaker J.J., Hill C.A.S., Eichhorn S.J., Bismarck A. (2011). Surface only modification of bacterial cellulose nanofibres with organic acids. Cellulose.

[61] Kokunešoski M., Gulicovski J., Matović B., Logar M., Milonjić S.K., Babić B. (2010). Synthesis and surface characterization of ordered mesoporous silica SBA-15. Mater. Chem. Phys..

[62] Shanks L.A., Cronshaw A., Alexander K.S., Davies J.A., O'Boyle C.P. (2020). Evaluation of EpiProtect® microbial cellulose burns dressings in young children. Scars Burn Heal..

[63] Queen D., Gaylor J.D.S., Evans J.H., Courtney J.M., Reid W.H. (1987). The preclinical evaluation of the water vapour transmission rate through burn wound dressings. Biomaterials.

[64] Rühs P.A., Malollari K.G., Binelli M.R., Crockett R., Balkenende D.W.R., Studart A.R., Messersmith P.B. (2020). Conformal bacterial cellulose coatings as lubricious surfaces. ACS Nano.

[65] Hou H., Zhao Y., Li C., Wang M., Xu X., Jin Y. (2017). Single-cell pH imaging and detection for pH profiling and label-free rapid identification of cancer-cells. Sci. Rep..

[66] Belotti Y., Jokhun D.S., Ponnambalam J.S., Valerio V.L.M., Lim C.T. (2021). Machine learning based approach to pH imaging and classification of single cancer cells. APL Bioengy.

[67] Buchan I.A., Andrews J.K., Lang S.M., Boorman J.G., V Harvey Kemble J., Lamberty B.G.H. (1981). Clinical and laboratory investigation of the composition and properties of human skin wound exudate under semi-permeable dressings. Burns.

[68] Vardaxis N.J., Brans T.A., Boon M.E., Kreis R.W., Marres L.M. (1997). Confocal laser scanning microscopy of porcine skin: implications for human wound healing studies. J. Anat..

