# Supplementary material for: Nanocellulose composite wound dressings for real-time pH wound monitoring
Source: Mater Today Bio. 2023 Feb 6;19:100574. doi: 10.1016/j.mtbio.2023.100574 (PMC9958357; doi:10.1016/j.mtbio.2023.100574)
Supplement: Multimedia component 1 [file mmc1.pdf]

Supplementary Information

Nanocellulose Composite Wound Dressings for Real-Time pH Wound Monitoring

Olof Eskilson[1,†], Elisa Zattarin[1,†], Linn Berglund[2], Kristiina Oksman[2], Kristina Hanna[3], Jonathan Rakar[3], Petter Sivlér[1], Mårten Skog[1], Ivana Rinklake[3], Rozalin Shamasha[3], Zeljana Sotra[3], Annika Starkenberg[3], Magnus Odén[4], Emanuel Wiman [5], Hazem Khalaf [5], Torbjörn Bengtsson[5], Johan P. E. Junker[3], Robert Selegård[1], Emma M. Björk[4], & Daniel Aili[1,]*

[1]Laboratory of Molecular Materials, Division of Biophysics and Bioengineering, Department of Physics, Chemistry and Biology, Linköping University, SE-581 83 Linköping, Sweden.

[2]Division of Materials Science, Department of Engineering Sciences and Mathematics, Luleå University of Technology, SE-971 87 Luleå, Sweden.

[3]Center for Disaster Medicine and Traumatology, Department of Biomedical and Clinical Sciences, Linköping University, SE-581 85 Linköping, Sweden.

[4]Division of Nanostructured Materials, Department of Physics, Chemistry and Biology (IFM), Linköping University, SE-58183 Linköping, Sweden.

[5]Cardiovascular Research Centre, School of Medical Sciences, Örebro University, SE-70362 Örebro, Sweden.

* Corresponding author: daniel.aili@liu.se

[†] Olof Eskilson and Elisa Zattarin contributed equally to this work

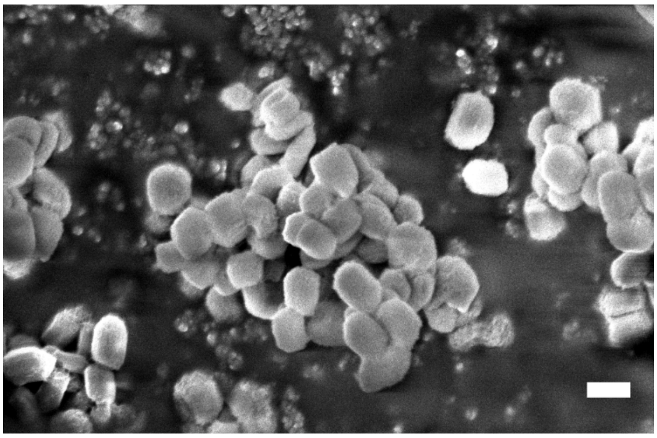

Figure S1. SEM micrograph of MSN. Scale bar: 400 nm.

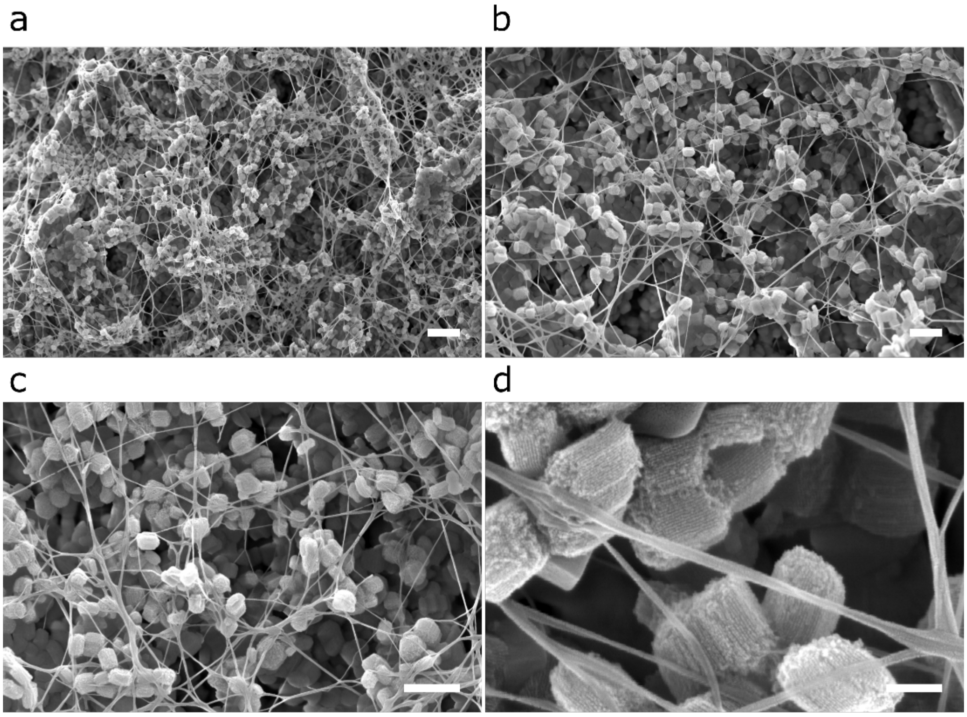

Figure S2. SEM micrographs of BC-MSN at different magnifications. a) Scale bar: 2 μm. b) Scale bar: 1 μm. c) Scale bar: 1 μm. d) Scale bar: 200 nm.

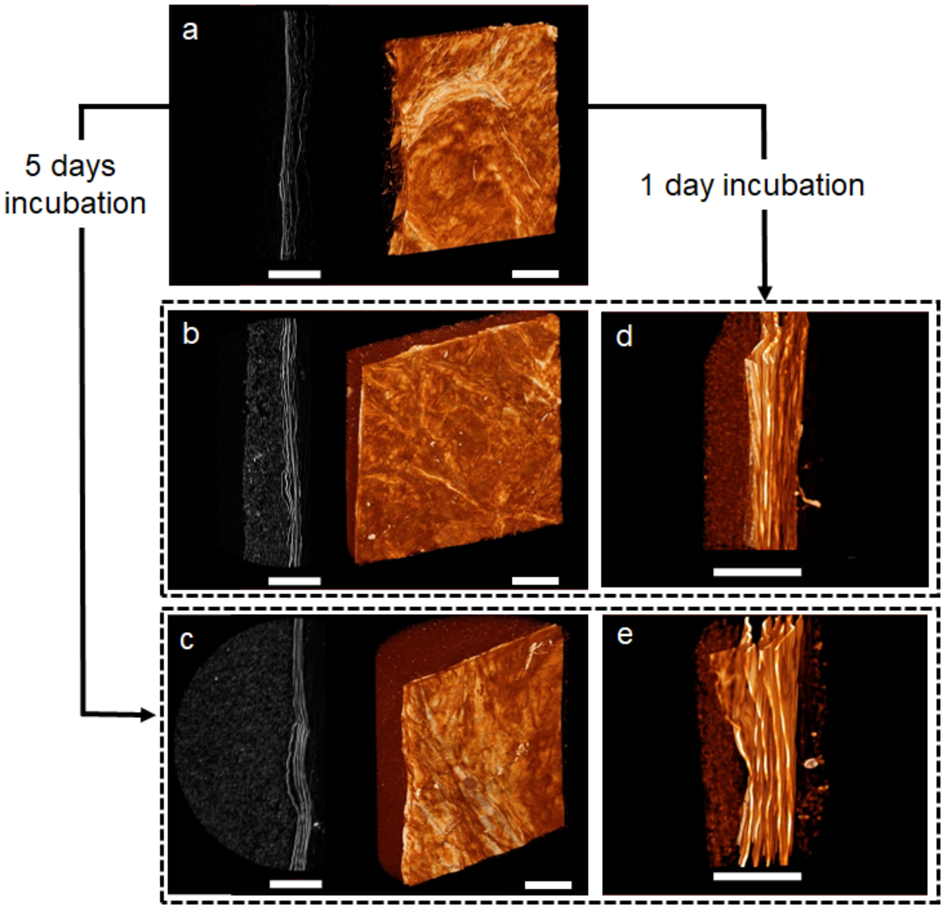

Figure S3. XRT reconstruction in 2D and 3D of a) BC, b) BC-MSN incubated in 5 mg/mL MSN for 1 day and c) BC-MSN incubated in 5 mg/mL MSN for 5 days visualized after freeze-drying. Scalebar: 100 µm. BC-MSN incubated in 5 mg/mL MSN for d) 1 day and e) 5 days, scalebar: 50 µm.

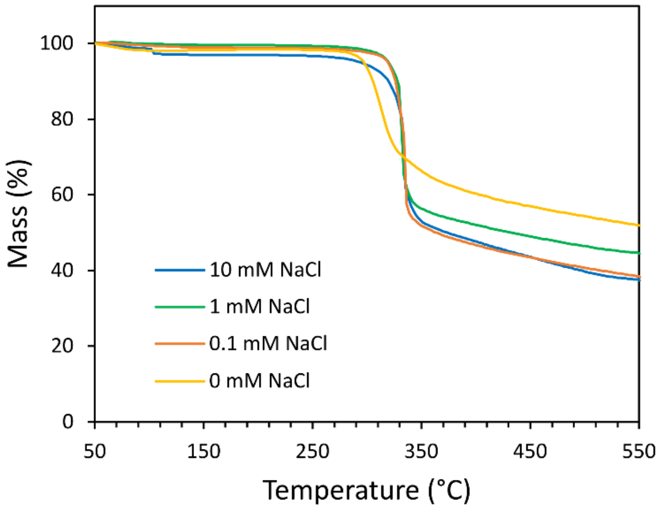

Figure S4. TGA of BC-MSN loaded in suspensions of different concentrations of NaCl.

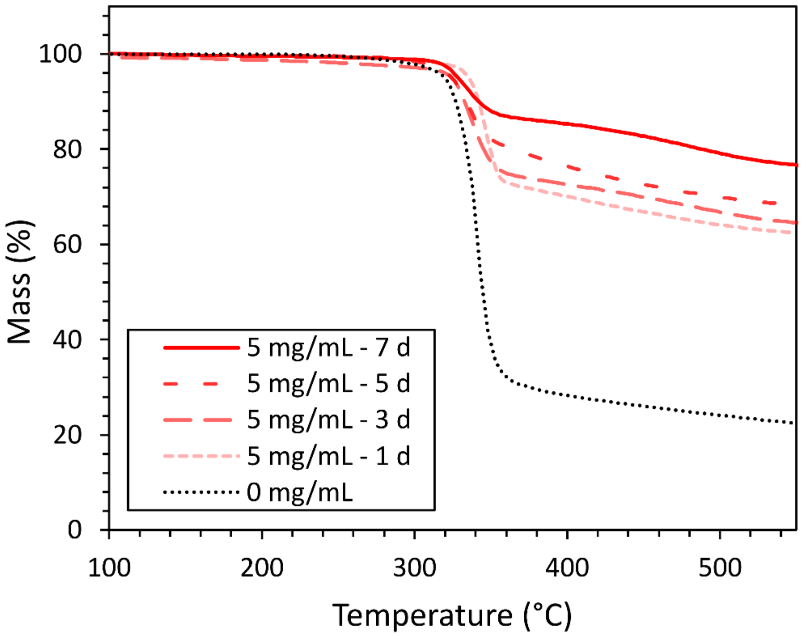

Figure S5. TGA curves of BC and BC-MSN composites incubated in 5 mg/mL MSN suspension for 1, 3, 5 and 7 days.

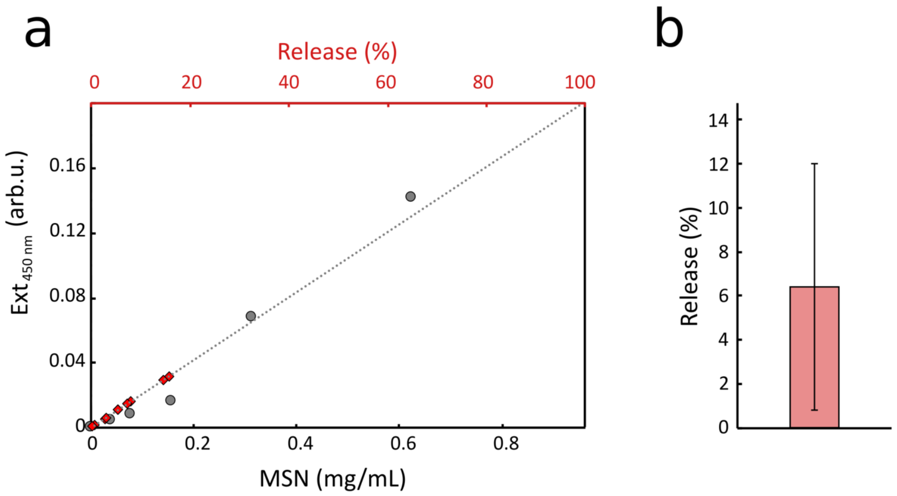

Figure S6. Detachment of MSNs from BC-MSNs after 3 days of shaking in PBS at room temperature. a) Calibration curve obtained measuring the extinction spectra at λ=450 nm of MSN suspension at different concentrations. Secondary x-axis indicates percentage MSN release in the defined volume. b) 6.4 ± 5.6 % of the MSNs were detached, n = 10.

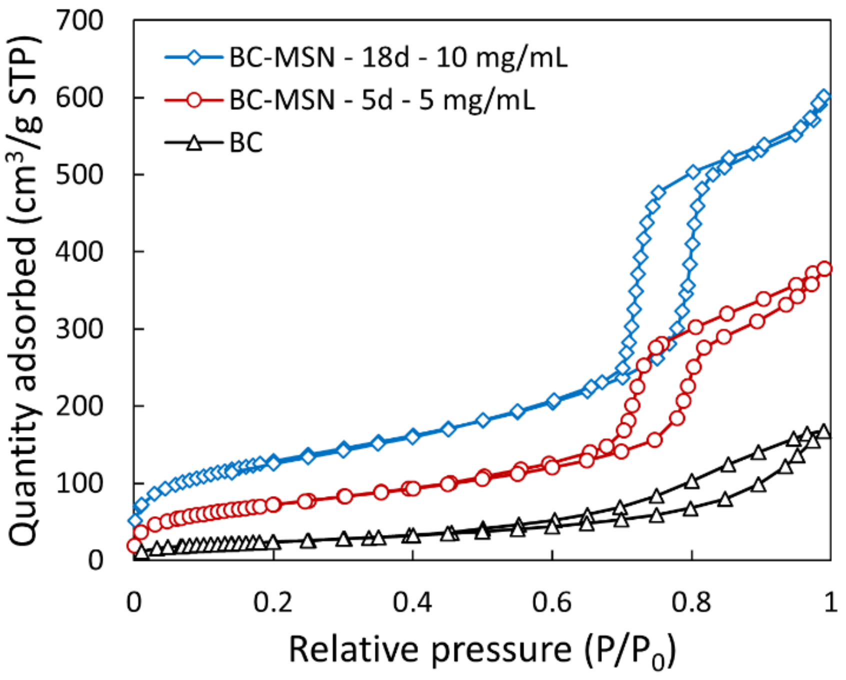

Figure S7. Nitrogen physisorption isotherms of BC and BC-MSN incubated in 5 mg/mL MSN for 5 days and 10 mg/mL for 18 days, respectively.

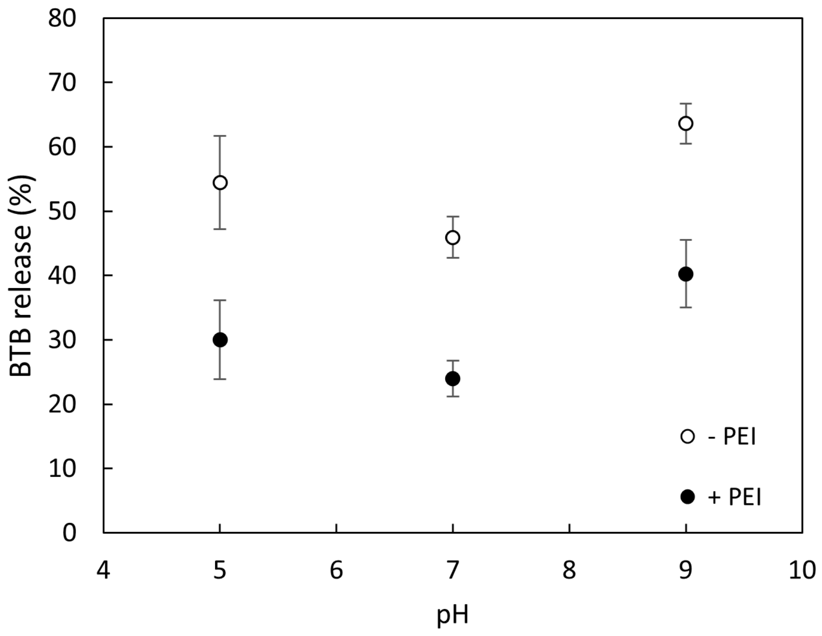

Figure S8. Effect of PEI capping on BTB retention on dressings washed in MTA buffer at pH 5, 7, and 9.

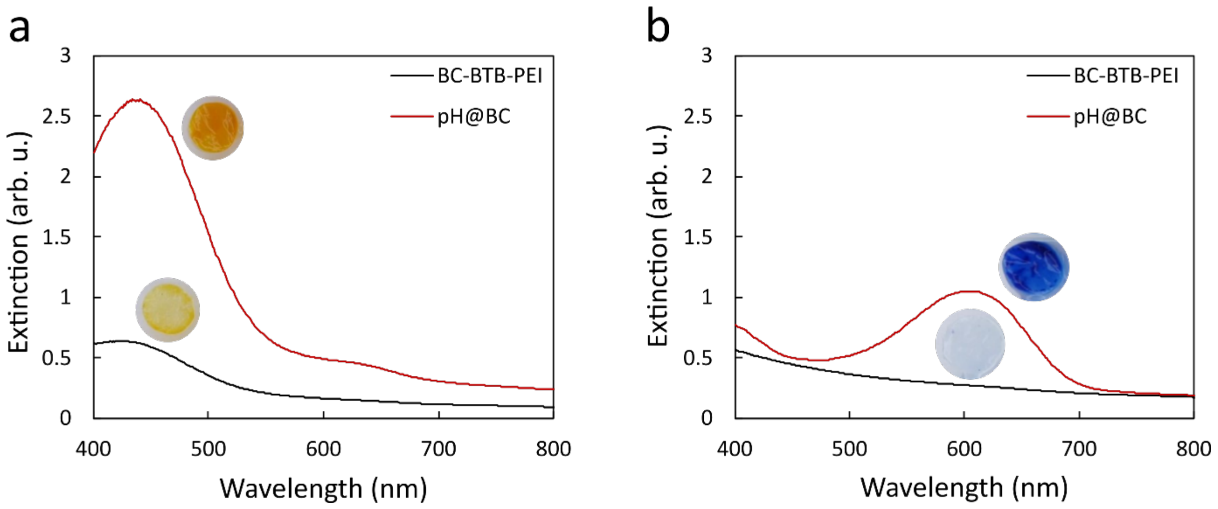

Figure S9. Effect of MSN on BTB loading and retention. Representative UV-vis spectra and photographs of a) freshly produced dressings (pH 5.5) and b) dressings subjected to 3 washing cycles in MTA buffer at pH 9.

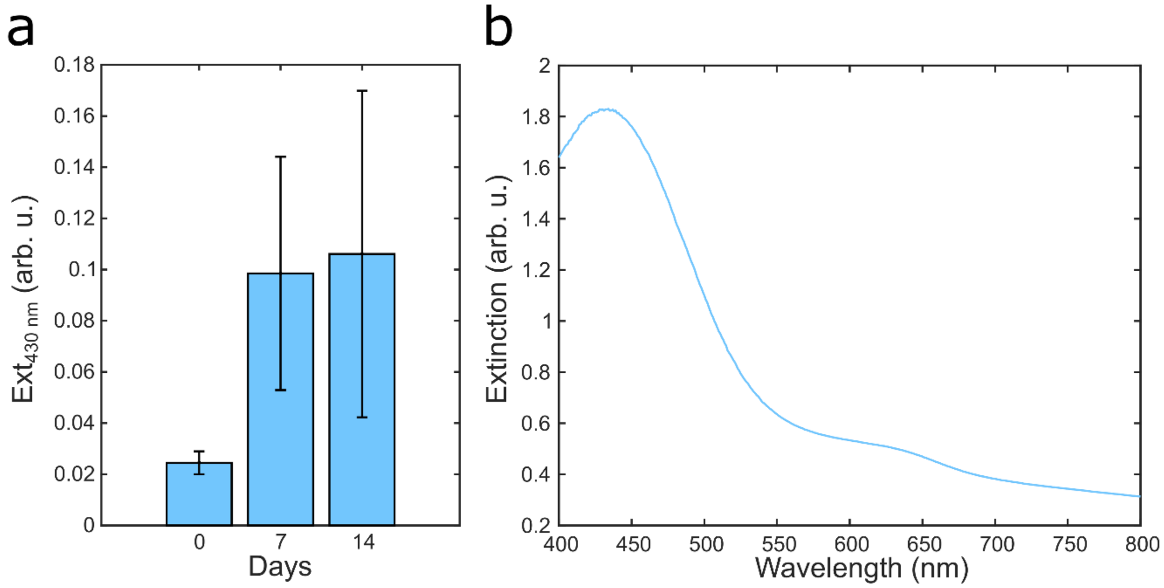

Figure S10. a) Extinction at λ = 430 nm of released BTB after incubating pH@BC for 0, 7 and 14 days in 1 mL of water (n = 3). b) Mean extinction of the pH@BC dressings after 14 days of incubation in water (1 mL), n=3.

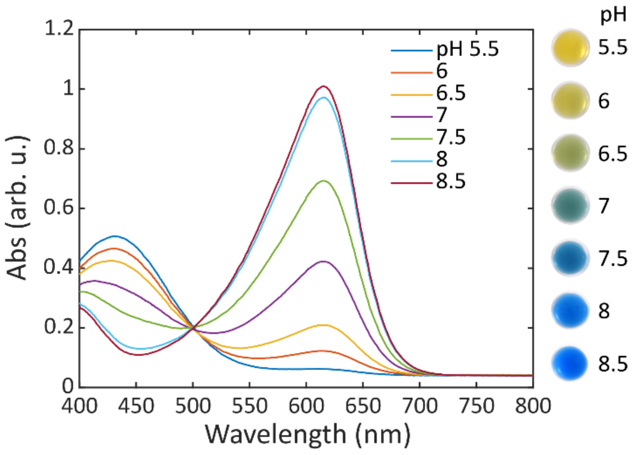

Figure S11. UV-vis spectra of BTB in aqueous buffers with different pH.

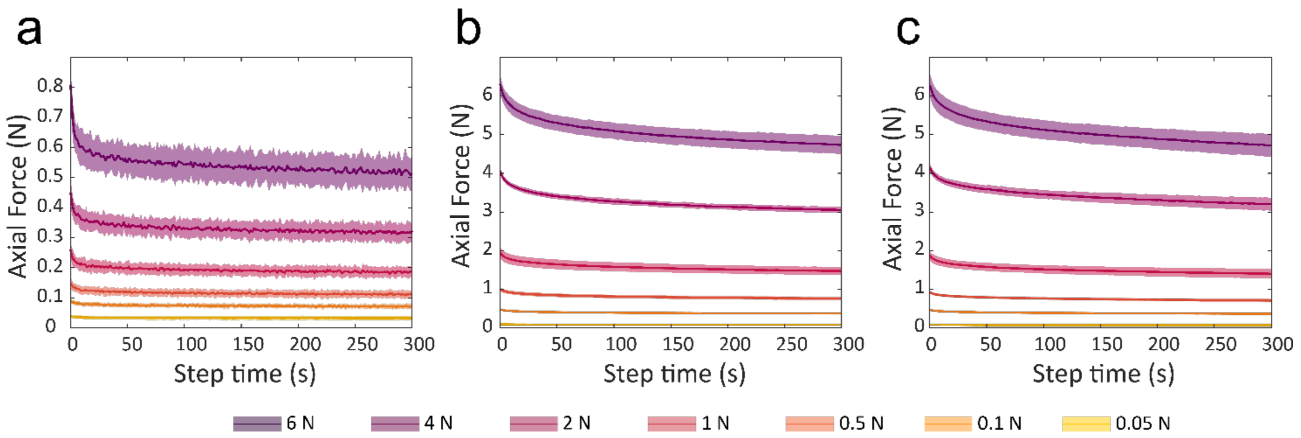

Figure S12. Stress relaxation of a) BC, b) BC-MSN and c) pH@BC following compression levels in the range 0.05 – 6 N, n=3.

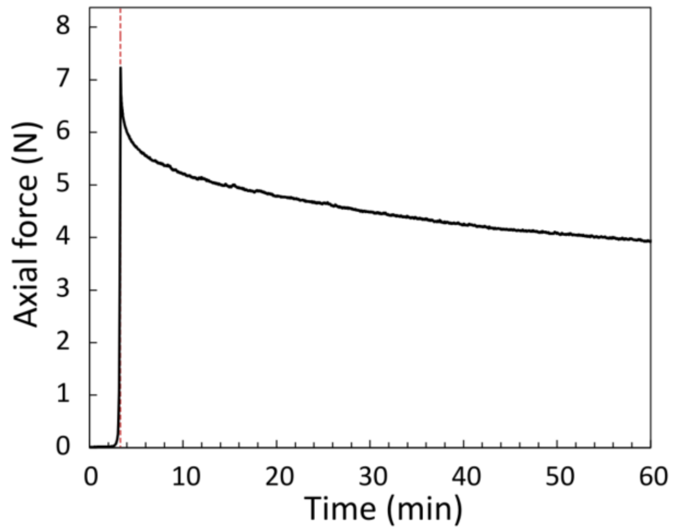

Figure S13. Relaxation of BC-MSN dressings subjected to ~ 7 N axial compression. The vertical line indicates the end of compression.

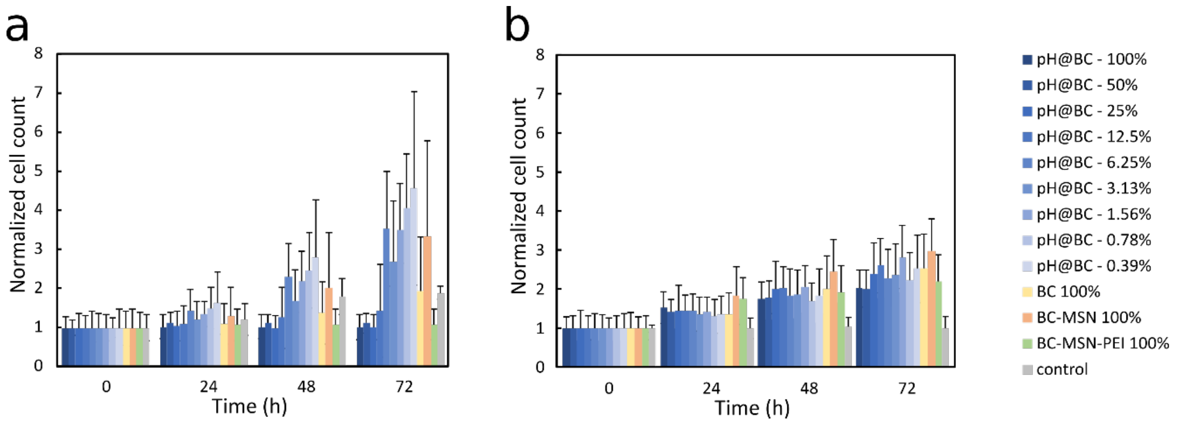

Figure S14. Cell proliferation of a) keratinocytes and b) fibroblasts when cultured in leachables from BC and the pH@BC dressings (n=8).

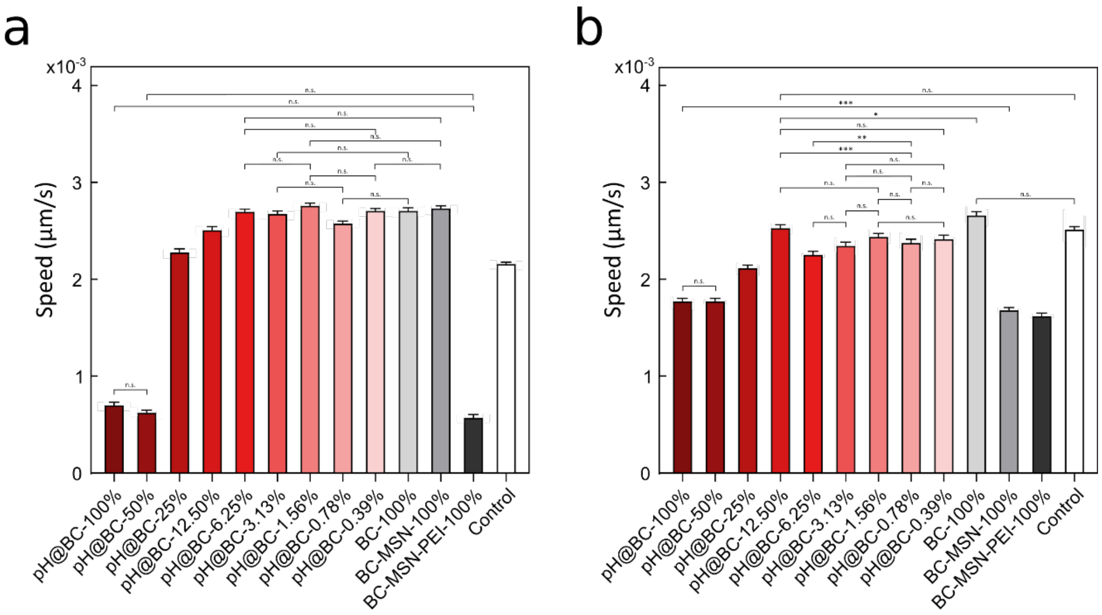

Figure S15. Migration rate of a) keratinocytes and b) fibroblasts when cultured in leachables from pH@BC. Results displayed as mean and standard error of the mean, * P < 0.1; ** P<0.01; *** P<0.001; P < 0.0001 whereas not indicated (n = 8).

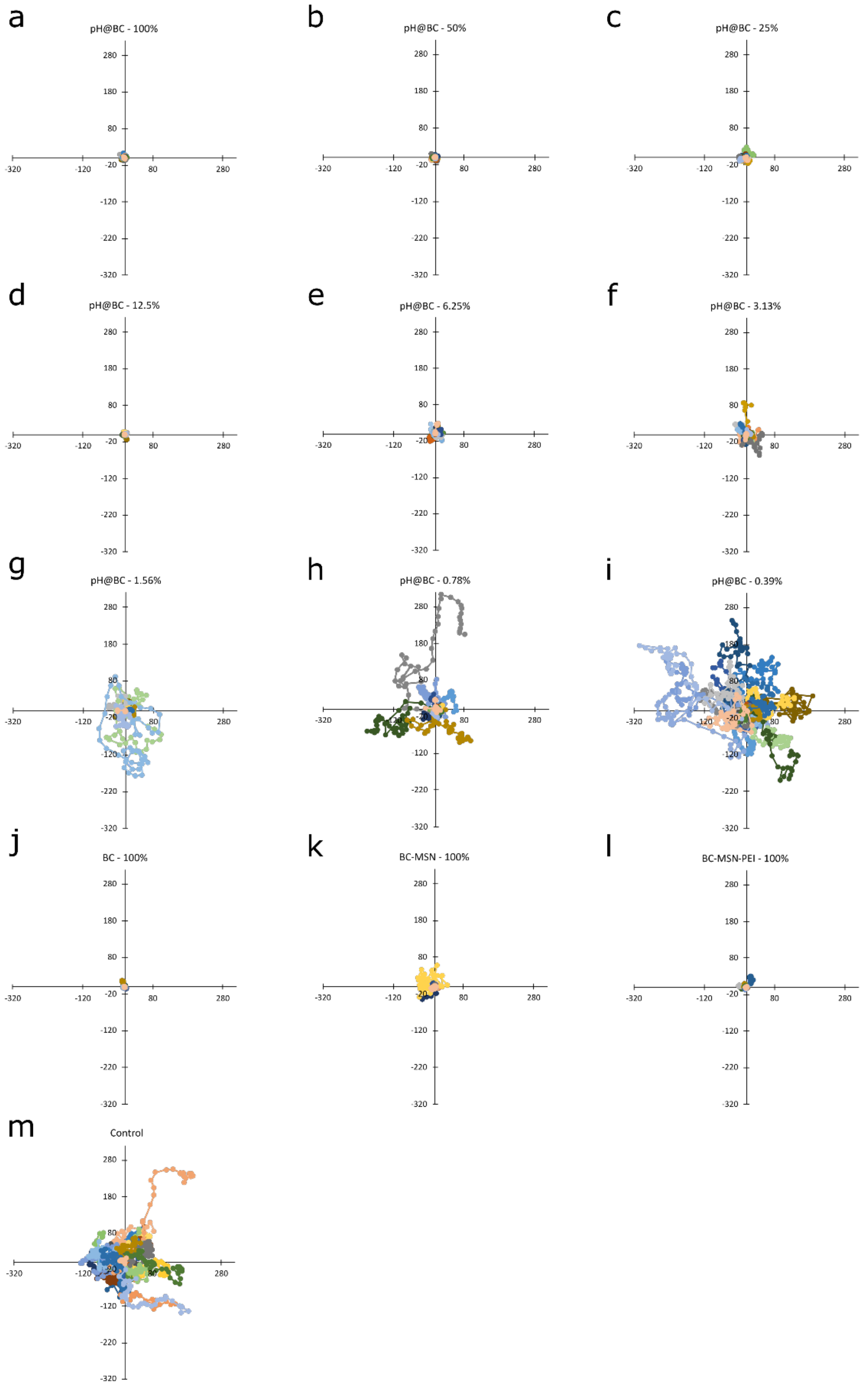

Figure S16. Keratinocyte motility measured over 72 h of a-i) pH@BC leachables 100%-0.39%, j) BC 100%, k) BC-MSN 100%, l) BC-MSN-PEI and, m) control samples.

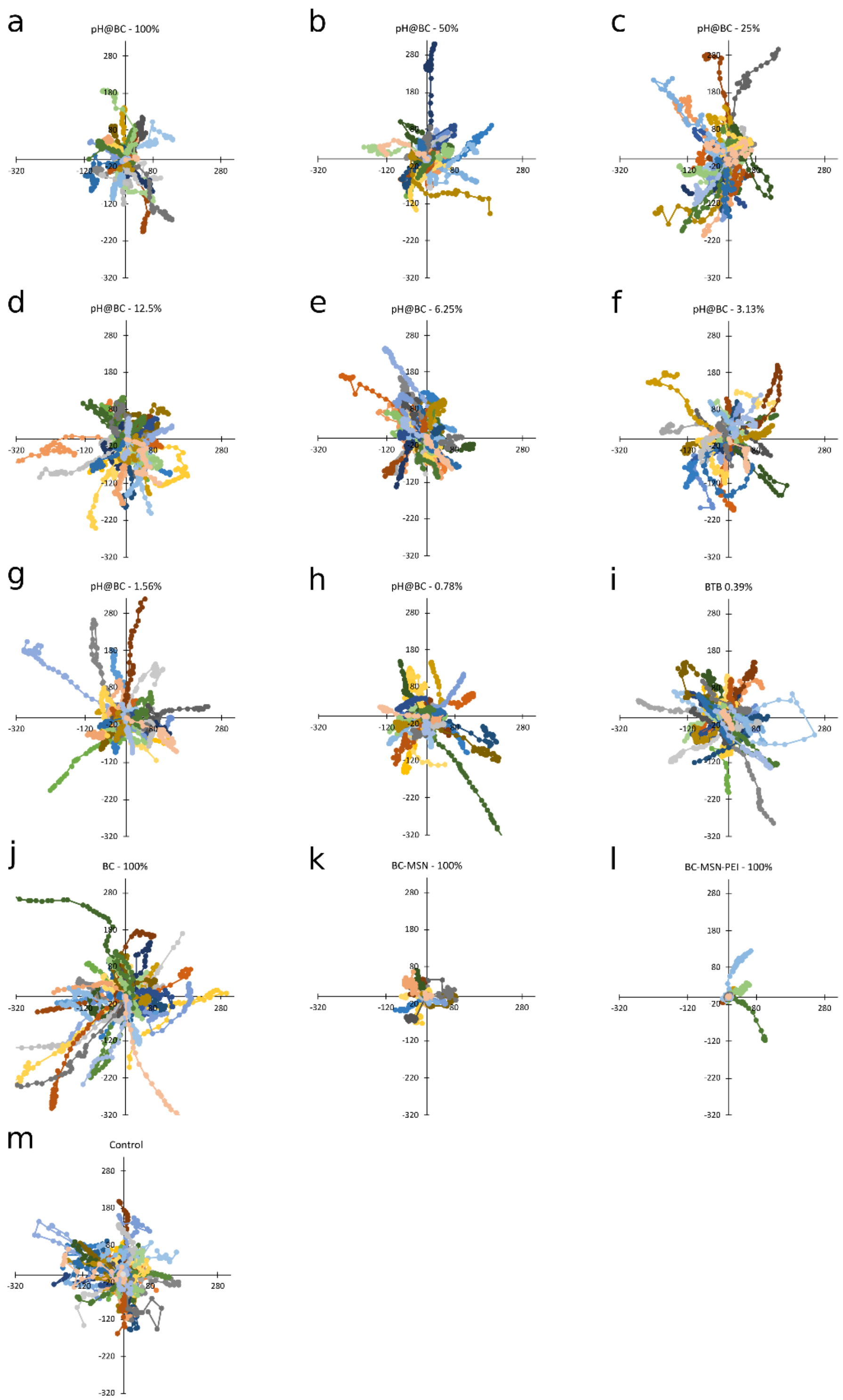

Figure S17. Fibroblast motility measured over 72 h of a-i) pH@BC leachables, 100%-0.39%, j) BC 100%, k) BC-MSN 100% and l) BC-MSN-PEI and, m) control samples.

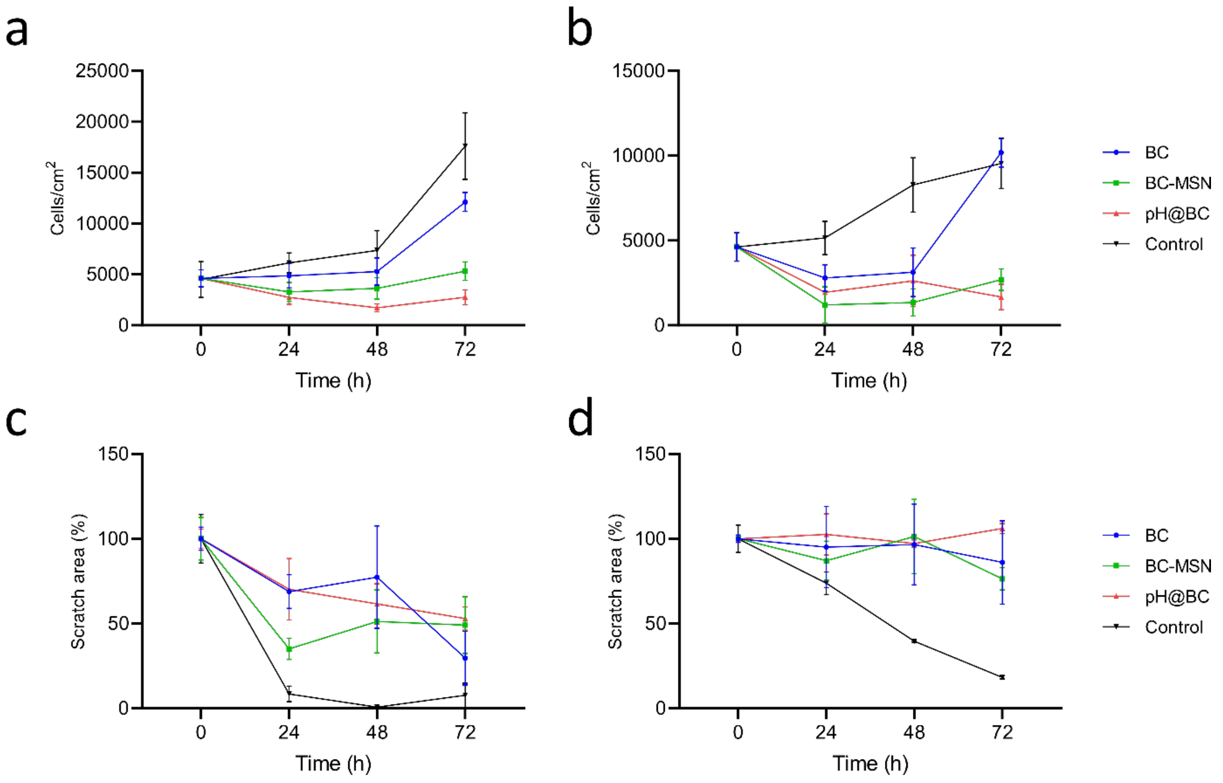

Figure S18. a) Fibroblast and b) keratinocyte proliferation when covered with membranes consisting of BC, BC-MSN, pH@BC and a non-covered control group. c) Fibroblast and d) keratinocyte scratch assay when covered with membranes consisting of BC, BC-MSN, pH@BC and a non-covered control group. Error bars show standard error of the mean (n = 3).

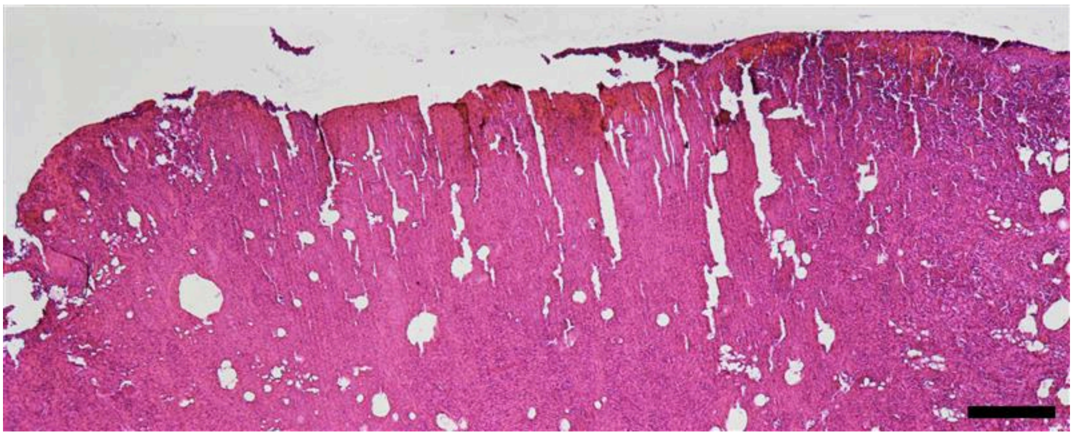

Figure S19. Histology of the infected porcine burn wound used for acquiring pH-sensing data reported in Fig. 5g. Staining with hematoxylin and eosin. Scalebar: 1 mm.
